# *GmRVE8a*, a circadian clock‐associated gene, negatively regulates flowering and maturity in soybean

**DOI:** 10.1111/tpj.71205

**Published:** 2026-11-04

**Authors:** Shibo Sun, Maoxiang Yang, Tingting Wu, Bingjun Jiang, Xuegang Sun, Hongchang Jia, Xiangyu Yao, Yanhui Sun, Qimeng Li, Peiguo Wang, Liwei Jiang, Chao Qin, Shan Yuan, Zhaoming Qi, Shi Sun, Huihui Li, Tianfu Han, Baiquan Sun

**Affiliations:** ^1^ College of Agriculture Northeast Agricultural University Harbin Heilongjiang 150030 China; ^2^ National Nanfan Research Institute, Chinese Academy of Agricultural Sciences Sanya Hainan 572024 China; ^3^ Ministry of Agriculture and Rural Affairs Key Laboratory of Soybean Biology (Beijing) Institute of Crop Sciences, Chinese Academy of Agricultural Sciences Beijing 100081 China; ^4^ Heihe Branch of Heilongjiang Academy of Agricultural Sciences Heihe Heilongjiang 164300 China

**Keywords:** soybean (*Glycine max* (L.) Merr.), genome‐wide association, *GmRVE8a*, circadian clock, flowering time, maturity

## Abstract

Precise regulation of flowering time and maturity is critical for achieving high and stable soybean yields, and the circadian clock plays a central role in coordinating these developmental transitions. Soybean is a typical short‐day crop with a pronounced photoperiodic response; however, the molecular mechanisms linking circadian regulation to photoperiod‐dependent flowering and maturity remain incompletely understood. Here, we integrated genome‐wide association analysis with deep learning approaches using 257 soybean germplasm accessions spanning 14 maturity groups and identified *GmRVE8a*, a homolog of the Arabidopsis circadian clock gene *REVEILLE*, as a strong candidate locus associated with soybean maturity across two field environments. Haplotype analysis demonstrated that accessions carrying the *GmRVE8a* ^*H1*^ haplotype consistently flowered and matured significantly later than those carrying *GmRVE8a* ^*H2*^. Functional validation revealed that knockout of *GmRVE8a* markedly accelerated flowering and maturity, whereas overexpression of *GmRVE8a* significantly delayed both processes. qRT‐PCR assays further suggested that *GmRVE8a* may repress flowering and delay maturity by promoting the expression of *GmFT4*, a flowering‐inhibitory member of the *FT* family, and is unlikely to act by directly regulating *E1* expression. Collectively, our findings suggest *GmRVE8a* as a circadian clock‐associated negative regulator of soybean flowering and maturity, and highlight it as a promising candidate for molecular breeding aimed at improving adaptation to diverse photoperiodic environments.

## Significance Statement

Flowering time and maturity determine where soybean can be grown and how reliably it produces yield. By integrating population genomics, deep learning, and functional validation, this study reveals *GmRVE8a* as a circadian clock‐associated regulator of soybean developmental timing, advancing our understanding of photoperiodic adaptation and providing a candidate target for crop improvement.

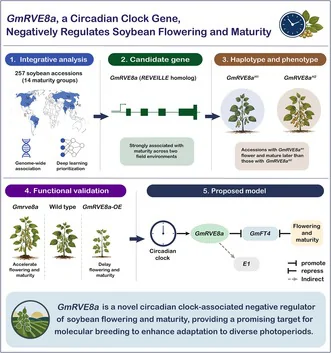

## INTRODUCTION

Soybean (*Glycine max* (L.) Merr.) is native to the temperate regions of China between 32° N and 40° N latitude and has since been introduced and cultivated worldwide, with its current cultivation range extending from 53° N to 35° S (Hymowitz, 1970; Hymowitz & Newell, 1981; Jiang et al., 2026; Zhang et al., 2020). Maturity is one of the most important ecological traits determining soybean adaptation and productivity, and is shaped by the integration of endogenous genetic regulatory networks with environmental cues, particularly photoperiod and temperature. As key components of photoperiod‐ and temperature‐responsive pathways, circadian clock‐related genes play central roles in coordinating these environmental signals and contribute substantially to the genetic diversity underlying soybean adaptation.

As a typical short‐day crop, soybean is highly sensitive to photoperiod, and its photoperiodic response and floral induction are major determinants of latitudinal adaptation and grain yield (Du et al., 2023). Accordingly, photoperiod sensitivity is a key trait regulating flowering time, maturity, and ecological adaptation in soybean. (Lin et al., 2021; Setiyono et al., 2007). To date, multiple genes regulating soybean flowering and maturity have been identified, including *GmRVE1*, *GmELF3* (*J*), *GmLUX1*, *GmLUX2*, *E2*, *E1*, *GmFT2a*, and *GmFT5a* (Cheng et al., 2026; Fang et al., 2024). Among these, *E1*, *E2*, *GmFT2a*, and *GmFT5a* contribute to soybean adaptation to high‐latitude regions (Dong et al., 2023; Wang et al., 2023; Xu et al., 2013, 2015), whereas *GmLUX1*, *GmLUX2*, and *J* facilitate soybean adaptation to low‐latitude regions (Dong et al., 2021; Kou et al., 2022; Yue et al., 2016). *E1*, a legume‐specific transcription factor, represses the expression of soybean *GmFT2a* and *GmFT5a*, and plays a dominant role in photoperiod‐regulated flowering and maturity (Kong et al., 2010). In turn, *E1* activity is repressed by *GmRVE1* (Cheng et al., 2026). *E2* encodes the soybean ortholog of Arabidopsis *GIGANTEA* (*GI*), a key component of the plant circadian clock (Wang et al., 2023). The *J* gene interacts with two homologs of LUX ARRHYTHMO to form the Evening Complex (EC), which represses *E1* expression and thus promotes soybean flowering (Bu et al., 2021). The EC–E1 pathway is widely recognized as a core regulatory module controlling photoperiodic flowering in soybean.

The circadian clock is an endogenous timekeeping system that enables organisms to anticipate and adapt to daily environmental fluctuations caused by the Earth's rotation (Li et al., 2025). In plants, the circadian system consists of interlocked transcription‐translation feedback loops (TTFLs) involving core clock components such as *TIMING OF CAB EXPRESSION 1*/*PSEUDO‐RESPONSE REGULATOR 1* (*TOC1*/*PRR1*), *CIRCADIAN CLOCK‐ASSOCIATED 1* (*CCA1*), *LATE ELONGATED HYPOCOTYL* (*LHY*), and members of the *REVEILLE* (*RVE*) family (Greenham & McClung, 2015). *TOC1*, a key member of the *PSEUDO‐RESPONSE REGULATOR* (*PRR*) gene family, was the first identified plant circadian regulator and peaks in expression at dusk (Laosuntisuk et al., 2023). Other *PRR* family members, including *PRR9*, *PRR7*, *PRR5*, and *PRR3*, are sequentially expressed from morning to evening and together form an essential component of the plant circadian oscillator (Li et al., 2025; Liu et al., 2026). *CCA1* and *LHY*, two MYB‐like transcription factors highly expressed at dawn, bind conserved promoter elements, including the Evening Element (EE) (Su et al., 2025) and the *CCA1* Binding Site (CBS) (Ni et al., 2009; Peng & Neff, 2020) to repress the transcription of *PRR* family genes. Conversely, sequentially expressed *PRR* proteins gradually repress *CCA1* and *LHY* expression from morning to night, forming the core negative feedback loop of the circadian clock.

RVEs represent another major group of plant circadian regulators and comprise a small subfamily of transcription factors harboring MYB domains. (Carré & Kim, 2002). In Arabidopsis, *RVE8* functions as a transcriptional activator that directly binds to EE motifs in the promoters of *PRR5*, *TOC1*, *LUX*, *ELF4*, and *GI* to promote their expression (Farinas & Mas, 2011; Rawat et al., 2011). *RVE4*, *RVE6*, and *RVE8* function in a partially redundant manner to accelerate the circadian rhythm. (Hsu et al., 2013). Notably, the *rve4 rve6 rve8* triple mutant exhibits a slightly delayed flowering phenotype, whereas the *rve8* single mutant shows the opposite effect, suggesting functional complexity and redundancy among *RVE* family members. At night, *TOC1* expression reaches its peak; it competes with RVEs for EE binding and indirectly promotes *CCA1* and *LHY* expression at the next dawn, closing the full circadian regulatory loop. However, among all soybean *RVE* family genes, only *GmRVE1* and *GmRVE5* have been functionally characterized to date. *GmRVE1* promotes soybean flowering and adaptation to high‐latitude regions by repressing *E1* transcription and thereby regulating *GmFT2a* and *GmFT5a* expression, whereas *GmRVE5* modulates the hypocotyl elongation of soybean (Cheng et al., 2026; Shen et al., 2025). The functions of other *RVE* family members remain to be further elucidated.

In this study, by integrating genome‐wide association study (GWAS) with DeepGOPlus‐based candidate gene prioritization, we implicated *GmRVE8a* as a putative suppressor of soybean flowering and maturity. We further validated its biological function through phenotypic and molecular characterization of CRISPR‐Cas9‐mediated knockout mutants and transgenic overexpression lines. Our results suggest that *GmRVE8a* may modulate soybean flowering time and maturity by promoting the expression of the floral repressor *GmFT4*. These findings reveal a previously uncharacterized role for *GmRVE8a* in soybean developmental timing and provide new insight into circadian regulation of photoperiodic adaptation.

## RESULTS

### Resequencing of soybean germplasm and genomic variation analysis

In this study, the Illumina NovaSeq sequencing platform was utilized to perform whole‐genome resequencing on 257 soybean germplasm accessions, with each accession generating high‐quality reads at an average sequencing depth of 9×. The resulting sequencing reads were mapped to the *Glycine max* Wm82.a2.v1 reference genome, covering approximately 94% of the genome (Sun et al., 2026; Table S1).

To characterize the genetic relatedness among the germplasm accessions, we applied a combination of complementary analytical approaches, encompassing principal component analysis (PCA; Figure S1) and phylogenetic reconstruction. To explore the genetic correlation characteristics among these germplasm accessions, multiple methods were comprehensively employed in this research, including principal component analysis (PCA; Figure S1) and phylogenetic analysis (Figure 1A). These analyses consistently clustered the 257 soybean accessions into three major groups, corresponding to the Northern Spring Soybean Planting Region, the Huang‐Huai‐Hai Summer Soybean Planting Region, and the Southern Multiple‐Cropping Soybean Planting Region of China. In addition, we analyzed the decay characteristics of linkage disequilibrium within the populations. The linkage disequilibrium strength, quantified by the *r* ^2^ value, showed a decreasing trend with the increase in physical distance between single‐nucleotide polymorphism (SNP) loci. The extent of linkage disequilibrium decay was estimated at roughly 50 kb, corresponding to the physical distance over which *r* ^2^ declined to 50% of its initial maximum (Figure S2).

**Figure 1:**
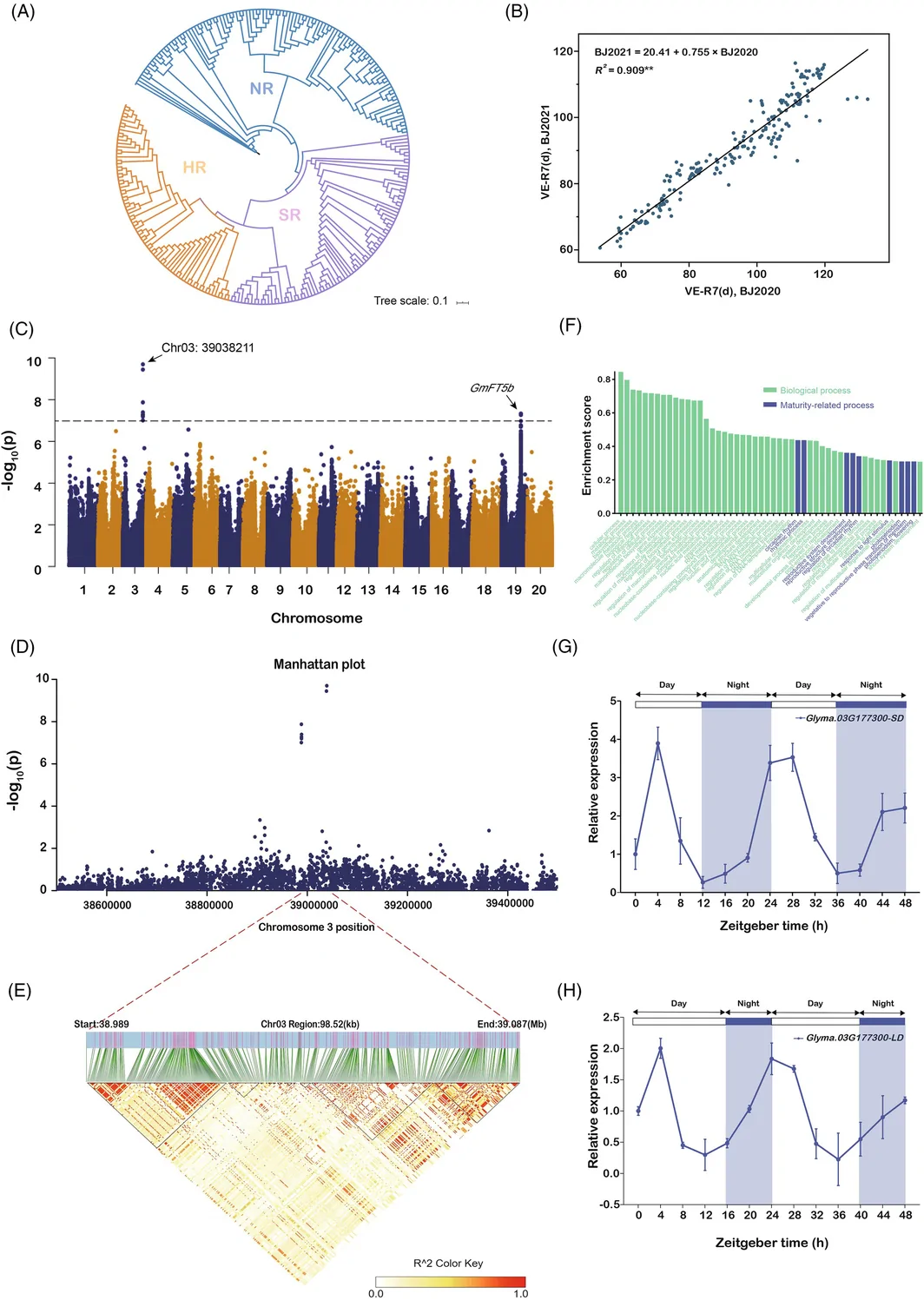
Identification of *GmRVE8a* locus. (A) Phylogenetic structure of the diversity panel based on analysis of genome‐wide SNPs. NR, accessions from the Northern Spring Planting Region; HR, accessions from the Huang–Huai–Hai Summer Planting Region; SR, accessions from the Southern Multiple Cropping Region. (B) Relationship between days from emergence to physiological maturity in BJ2020 and BJ2021. VE, emergence; R7, physiological maturity; VE‐R7, days from VE to R7; d, day. BJ2020, Beijing (40°23′ N, 116°57′ E), China, in 2020; BJ2021, Beijing, China, in 2021. (C) Genome‐wide association study result for VE–R7. Dashed horizontal lines indicate the Bonferroni‐corrected significance threshold. Candidate loci and previously reported flowering‐ or maturity‐related gene are annotated. (D) Local Manhattan plots for VE–R7. (E) Heatmap surrounding the candidate region for the peak. Red line indicates the location of Chr03: 39038211. Black triangle indicates the linkage disequilibrium block surrounding Chr03: 39038211. (F) Gene ontology (GO) enrichment scores for biological process (BP) in the candidate genomic region linked to soybean maturity, calculated using the deep learning‐based DeepGOPlus model. (G, H) Diurnal expression profiles of *Glyma.03G177300* under short‐day conditions, SD, 12 h light/12 h dark, and long‐day conditions, LD, 16 h light/8 h dark. Data are shown as mean ± standard deviation from three independent biological replicates.

### GWAS mapping of maturity‐associated QTLs

Phenotypic distribution analysis showed that maturity trait VE‐R7 exhibited continuous variation among soybean varieties and followed an approximately normal distribution (Figure S3). Phenotypic data for maturity (VE‐R7) were compared between BJ2020 and BJ2021. The trait showed high consistency across the 2 years (*R* ^2^ = 0.909), indicating strong phenotypic stability under the tested environments. To dissect the genetic regulatory pathways underlying soybean maturity, GWAS was employed for in‐depth analysis, leading to the identification of a consistently significant QTL region on Chr.03 (−log_10_ *P* ≥ 7) (Figure 1C,D, Figure S4 and Table S2). Additionally, other QTL loci potentially associated with the known gene *GmFT5b* were detected on Chr.19, supporting the reliability of the GWAS model in capturing known phenotype‐associated loci.

To define the candidate region on Chr.03, we further investigated the local linkage disequilibrium structure surrounding the major associated SNPs based on the estimated genome‐wide linkage disequilibrium decay distance of approximately 50 kb. Linkage disequilibrium block structure analysis revealed that the candidate genomic region was delimited to a 98‐kb interval on Chr.03, spanning 38.989–39.087 Mb (Figure 1E). According to the annotation of the soybean reference genome *Glycine max* Wm82.a2.v1, this interval harbored nine annotated genes (Table S3).

### Identification of genetic loci regulating maturity

DeepGOPlus (Yang et al., 2023) was employed to screen and prioritize candidate genes within the target interval. Gene Ontology (GO) term prediction prioritized *Glyma.03G177300* as the most relevant candidate gene associated with flowering‐ and maturity‐related biological processes, including circadian rhythm and photoperiodism (Figure 1F and Table S4). Accordingly, we used quantitative real‐time PCR (qRT‐PCR) to characterize the expression profile of *Glyma.03G177300* in the soybean cultivar Jack. Under both short‐day (SD; 12 h light/12 h dark) and long‐day (LD; 16 h light/8 h dark) photoperiods, *Glyma.03G177300* displayed a pronounced oscillatory expression profile, implying that its transcription may be governed by the circadian clock (Figure 1G,H). Further analysis showed that *Glyma.03G177300* was highly expressed in floral organs, with moderate expression in trifoliate leaves and true leaves (Figure S5a,b), tissues that are crucial for soybean perception of external photoperiod signals. We next performed multiple sequence alignment of the encoded protein with *RVE8* homologs from Arabidopsis and other crop species (Figure S6). This analysis revealed that *Glyma.03G177300* contains the hallmark MYB DNA‐binding domain and critical functional residues characteristic of the *RVE8* subfamily, and shows high sequence conservation with known *RVE8* homologs. Based on these findings, we designated *Glyma.03G177300* as *GmRVE8a*.

### Natural variation and haplotype characterization of *GmRVE8a*

To elucidate the biological role of *GmRVE8a*, we characterized its SNPs and insertions/deletions (InDels), identifying eight SNPs within the coding sequence (CDS) (Figure 2A).

**Figure 2:**
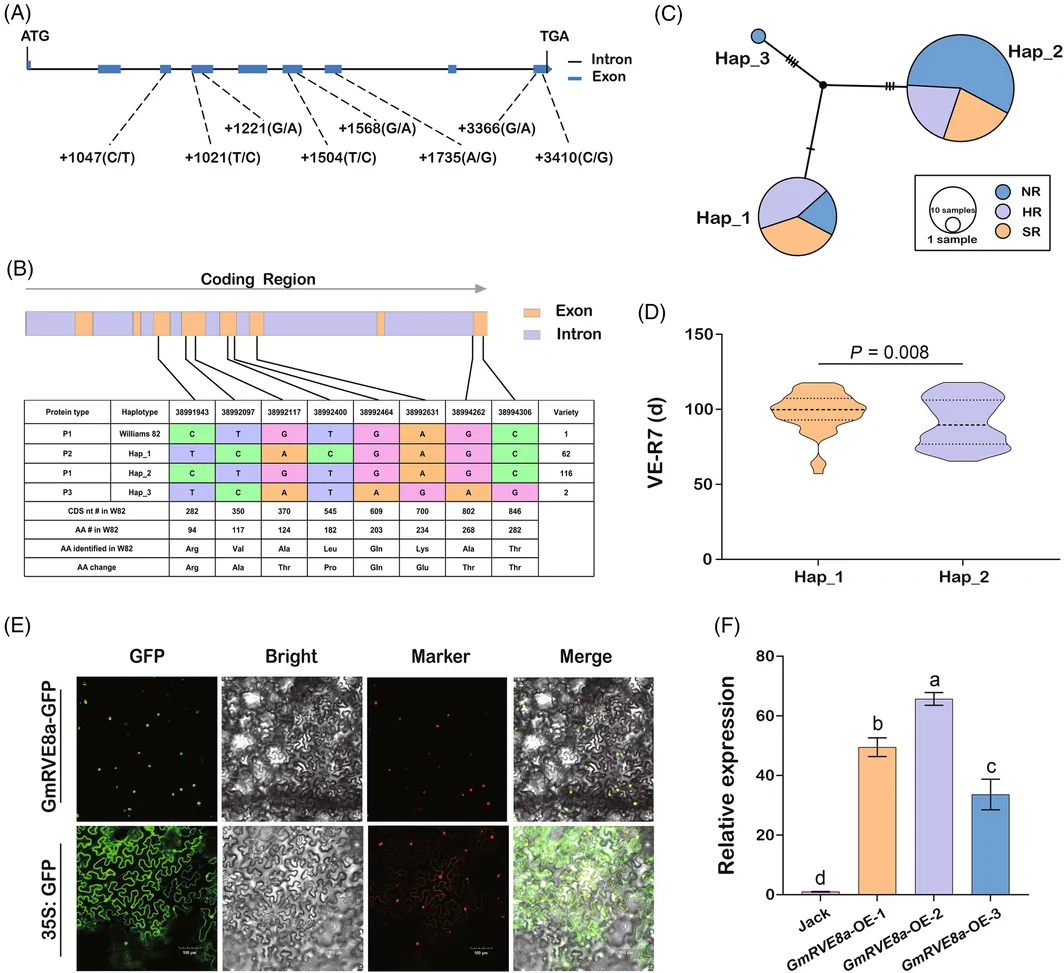
Molecular characterization and functional analysis of *GmRVE8a*. (A) Gene structure and coding region variations of *GmRVE8a* in different haplotypes. Exons and introns are indicated. ATG and TGA denote the translation start and stop codons. (B) Haplotypes detected in the coding region of *GmRVE8a* and their predicted amino acid changes. W82 refers to Williams 82, the soybean reference cultivar. CDS, coding sequence; AA, amino acid. # denotes nucleotide or amino acid positions. (C) Haplotype network of *GmRVE8a* based on coding region variants. Circle size is proportional to the number of accessions carrying each haplotype. Colors indicate soybean accessions from different ecological regions: NR, Northern Spring Planting Region; HR, Huang–Huai–Hai Summer Planting Region; SR, Southern Multiple Cropping Region. Short bars on branches indicate mutational steps between haplotypes. (D) Comparison of maturity between the major haplotypes *GmRVE8a* ^*H1*^ and *GmRVE8a* ^*H2*^. VE, emergence; R7, physiological maturity; VE‐R7, days from VE to R7; d, day. Statistical significance was determined using a two‐sided Student's t test. (E) Subcellular localization of GmRVE8a‐GFP. *Agrobacterium tumefaciens*‐mediated transient expression was performed to introduce the 35S: GmRVE8a‐GFP fusion construct and 35S: GFP empty vector (negative control) into *N. benthamiana* leaves. The 35S: mCherry‐NLS construct was co‐transformed as a specific nuclear localization marker. GFP fluorescence (green), bright‐field images, mCherry fluorescence (red, nuclear marker), and merged images are presented from left to right. Yellow signals in the merged panels indicate co‐localization of GFP fusion proteins with the nuclear marker. Scale bar: 100 μm. (F) Identification of *GmRVE8a*‐overexpression (*GmRVE8a*‐OE) transgenic soybean lines. Transcript levels of *GmRVE8a* in the wild‐type Jack and three independent *GmRVE8a*‐OE transgenic lines were detected by qRT‐PCR. All three overexpression lines showed significantly higher *GmRVE8a* expression levels compared with the wild‐type Jack. Error bars represent the standard deviation of three independent biological replicates. Different lowercase letters indicate statistically significant differences at the *P* < 0.05 level as determined by Duncan's multiple range test.

Haplotype analysis revealed three distinct haplotypes (Figure 2B). Among these, *GmRVE8a* ^*H1*^ was commonly observed in the southern regions (SR), whereas *GmRVE8a* ^*H2*^ was predominantly found in the northern region (NR) (Figure 2C), *GmRVE8a* ^*H3*^, with low sample sizes (*n* < 5), was not further investigated.

Across the two tested environments, accessions carrying the *GmRVE8a* ^*H1*^ haplotype exhibited significantly later maturity than those carrying *GmRVE8a* ^*H2*^ (*P* < 0.01) (Figure 2D). The two haplotypes differ by several nonsynonymous variants, resulting in amino acid substitutions at positions 117 (Val to Ala), 124 (Ala to Thr), and 182 (Leu to Pro) (Figure 2B). Collectively, these findings suggest that *GmRVE8a* represents a strong candidate gene underlying natural variation in soybean maturity.

### Subcellular localization of *GmRVE8a*

To further explore the molecular characteristics of *GmRVE8a*, we performed its subcellular localization analysis. Confocal microscopy showed that the GmRVE8a‐GFP fusion protein was exclusively localized in the nucleus (Figure 2E). Nuclear localization is a prerequisite for transcription factors to bind downstream target genes and regulate transcription, supporting the hypothesis that *GmRVE8a* functions as a transcription factor involved in circadian regulation in soybean.

### CRISPR‐Cas9‐mediated *Gmrve8a* mutants display early flowering time and maturity

To further substantiate the involvement of *GmRVE8a* in the regulation of flowering time and maturity, we generated *Gmrve8a* loss‐of‐function mutants in the Williams 82 cultivar (Wm82) genetic background using CRISPR‐Cas9 technology. Two homozygous mutant lines were identified and selected for subsequent phenotypic analyses (Figure S7).

Under SD conditions, both independent mutant lines exhibited significantly earlier flowering than Wm82, with flowering occurring 4–5 days earlier (*P* < 0.05). Their maturity was also markedly accelerated by 3–4 days (*P* < 0.05; Figure S8a,b). Under LD conditions, the mutant lines flowered 7–8 days earlier and reached maturity 9–10 days earlier than Wm82 (*P* < 0.05; Figure S8c,d).

Under natural long‐day (NLD) conditions in Beijing from June to October 2023, both independent mutant lines exhibited earlier flowering than Wm82, with flowering occurring 6–7 days sooner (*P* < 0.05; Figure 3A,B), maturity was likewise attained 4–5 days earlier in the mutant lines (*P* < 0.05; Figure 3A,D). Consistent with these observations, plants of both mutant lines grown under NLD conditions in Xinxiang in 2023 flowered 1–2 days earlier and reached maturity 6–7 days earlier than Wm82 (*P* < 0.05; Figure 3F,H). Consistent results were obtained in replicate field experiments conducted in 2025 (Figure 3C,E,G,I). Taken together, these findings suggest that loss of *GmRVE8a* function promotes flowering and maturation across diverse photoperiodic environments.

**Figure 3:**
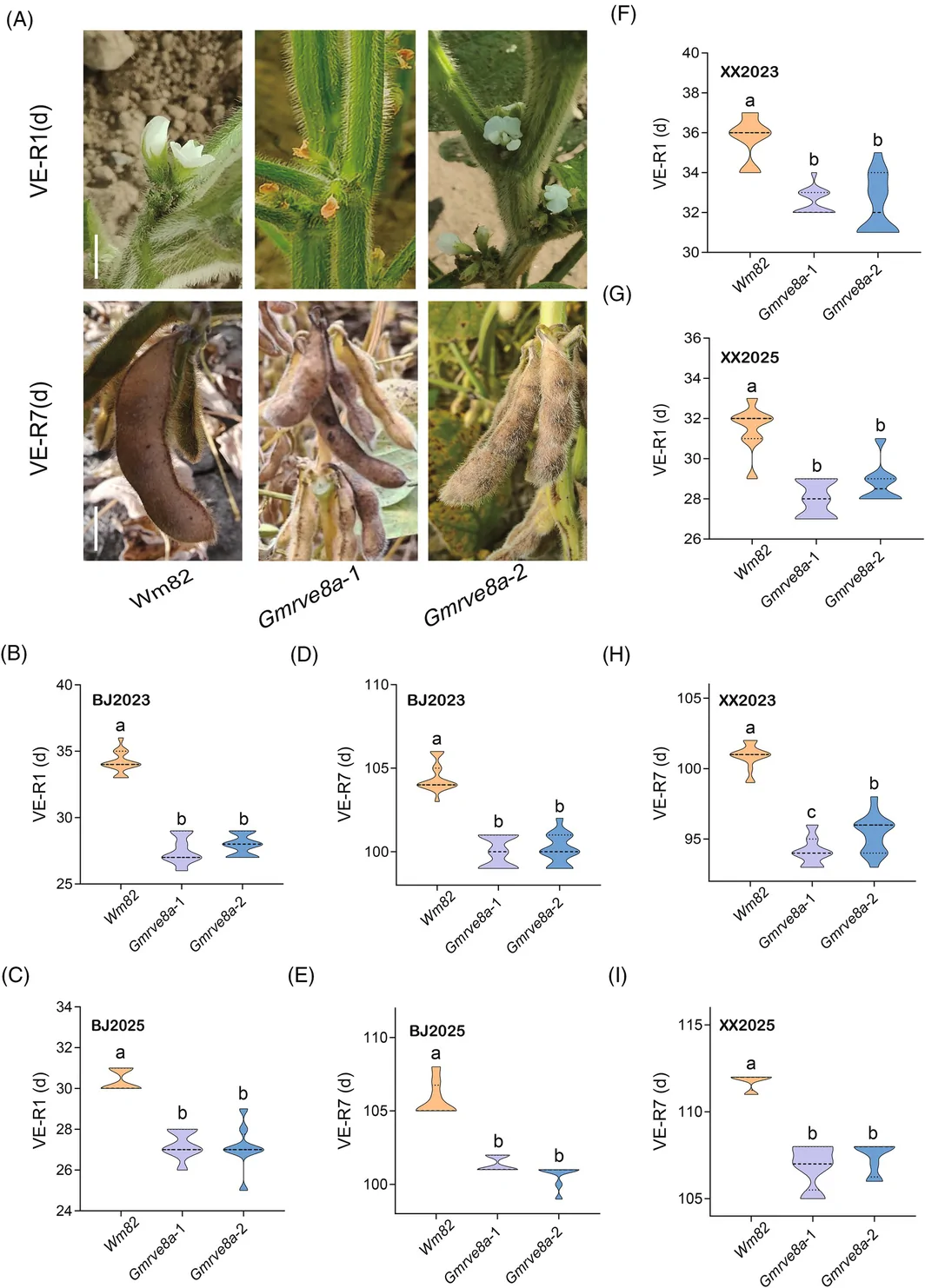
Phenotypic characterization of *Gmrve8a*‐mutant lines. (A) Plant phenotypes related to flowering time and maturity under natural long‐day (NLD) conditions. (B, C, F, G) Flowering time (days from VE to R1). VE, emergence; R1, beginning bloom. (D, E, H, I) Maturity (days from VE to R7). R7, physiological maturity. n = 12 plants. d, day. Wm82, Williams 82. BJ2023, Beijing (40°23′ N, 116°57′ E), China, in 2023; BJ2025, Beijing, China, in 2025. XX2023, Xinxiang (35°10′ N, 113°48′ E), China, in 2023; XX2025, Xinxiang, China, in 2025. Flowering stage scale bars: 3 cm. Maturity scale bars: 5 cm. Different lowercase letters indicate statistically significant differences at the *P* < 0.05 level as determined by Duncan's multiple range test.

### Overexpression of *GmRVE8a* in soybean delays flowering time and maturity

To further validate the role of *GmRVE8a* in regulating flowering time and maturity, we generated a *GmRVE8a* ^*H1*^ overexpression construct by placing its coding sequence under the control of the constitutive *CaMV35S* promoter, and subsequently introduced the construct into the soybean cultivar Jack via genetic transformation. qRT‐PCR analysis confirmed markedly increased *GmRVE8a* transcript levels in three independent T_3_ transgenic lines (*GmRVE8a*‐OE lines), with 49‐, 65‐, and 33‐fold higher expression than in Jack, respectively (Figure 2F).

Under SD conditions, the *GmRVE8a*‐OE lines flowered 3–4 days later than Jack and reached maturity 5–6 days later (*P* < 0.05; Figure S8e,f). Under LD conditions, flowering and maturity were postponed by 8–9 and 11–12 days, respectively, relative to Jack (*P* < 0.05; Figure S8g,h).

When grown under NLD conditions in Beijing in 2023, the *GmRVE8a*‐OE lines displayed 6–7 days delay in flowering and 7–8 days delay in maturity compared with Jack (*P* < 0.05; Figure 4A,B,D). Consistent with these results, the *GmRVE8a*‐OE lines flowered 7–9 days later and matured 7–8 days later than Jack under NLD conditions in Xinxiang in 2023 (*P* < 0.05; Figure 4F,H). Consistent results were obtained in replicate field experiments conducted in 2025 (Figure 4C,E,G,I). Collectively, these findings suggest that *GmRVE8a* delays flowering and maturity across diverse photoperiodic environments.

**Figure 4:**
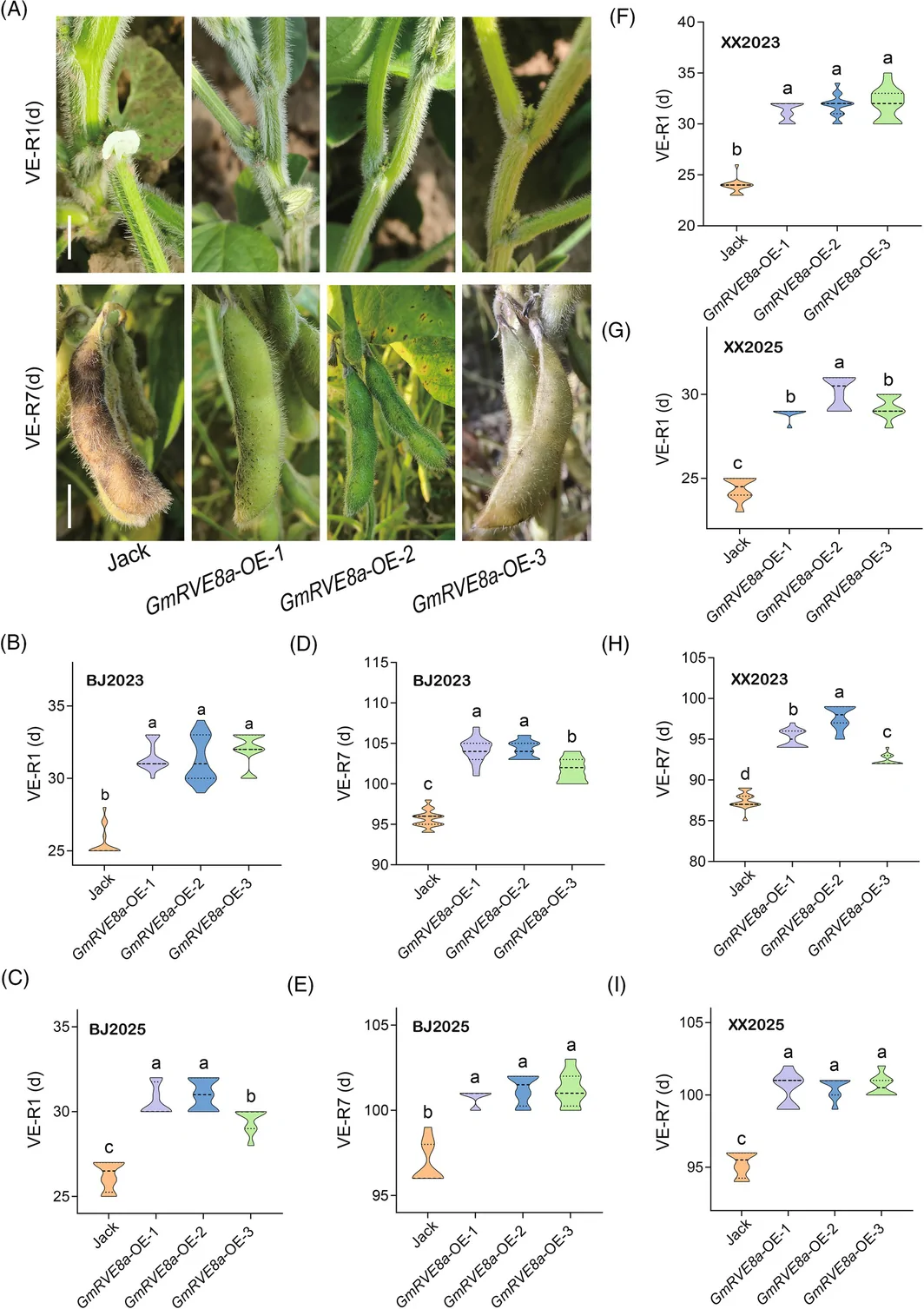
Phenotypic characterization of *GmRVE8a*‐overexpression (*GmRVE8a*‐OE) transgenic soybean lines. (A) Plant phenotypes related to flowering time and maturity under natural long‐day (NLD) conditions. (B, C, F, G) Flowering time (days from VE to R1). VE, emergence; R1, beginning bloom. (D, E, H, I) Maturity (days from VE to R7). R7, physiological maturity. *n* = 12 plants. d, day. BJ2023, Beijing (40°23′ N, 116°57′ E), China, in 2023; BJ2025, Beijing, China, in 2025. XX2023, Xinxiang (35°10′ N, 113°48′ E), China, in 2023; XX2025, Xinxiang, China, in 2025. Flowering stage scale bars: 3 cm. Maturity scale bars: 5 cm. Different lowercase letters indicate statistically significant differences at the *P* < 0.05 level as determined by Duncan's multiple range test.

### *GmRVE8a* may exert its function by positively regulating *GmFT4* expression

To further investigate the relationship between *GmRVE8a* and photoperiod flowering‐related genes, and to account for the rhythmic expression characteristics of flowering‐time regulators, we performed a 48‐h time‐course expression analysis under both LD and SD conditions. The expression profiles of eight key genes involved in the soybean photoperiod flowering pathway, including *E1*, *GmE1La*, *GmE1Lb*, *GmFT4*, *GmFT5a*, *GmFT2b*, *GmELF3*, and *GmLUX*, were examined in *Gmrve8a*, *GmRVE8a*‐OE, Jack, and Wm82 lines.

The 48‐h time‐course expression analysis showed that *GmFT4* transcript levels were consistently reduced in *Gmrve8a* mutants compared with Wm82, but consistently increased in *GmRVE8a*‐OE lines compared with Jack at multiple time points under both LD and SD conditions (Figure 5D,L; Figure S9d,l). This reciprocal expression pattern suggests that *GmRVE8a* may promote, or be positively associated with, *GmFT4* expression. By contrast, *E1* did not show a consistent expression response associated with altered *GmRVE8a* activity under either LD or SD conditions (Figure 5A,I; Figure S9a,i). Similar patterns were observed for the *E1* homologs *GmE1La* and *GmE1Lb* (Figure 5B,C,J,K; Figure S9b,c,j,k), suggesting that *GmRVE8a* is unlikely to regulate flowering mainly through direct activation of *E1*. Other flowering‐ and circadian‐related genes, including *GmFT5a*, *GmFT2b*, *GmELF3*, and *GmLUX*, displayed genotype‐ and photoperiod‐dependent expression changes during the 48‐h time course (Figure 5E–H,M–P; Figure S9e–h,m–p). Nevertheless, among the examined genes, *GmFT4* showed the most consistent reciprocal response to *GmRVE8a* loss‐ and gain‐of‐function under both LD and SD conditions. These results suggest that *GmRVE8a* may exert its function by positively regulating *GmFT4* expression and is unlikely to act by directly regulating *E1* expression.

**Figure 5:**
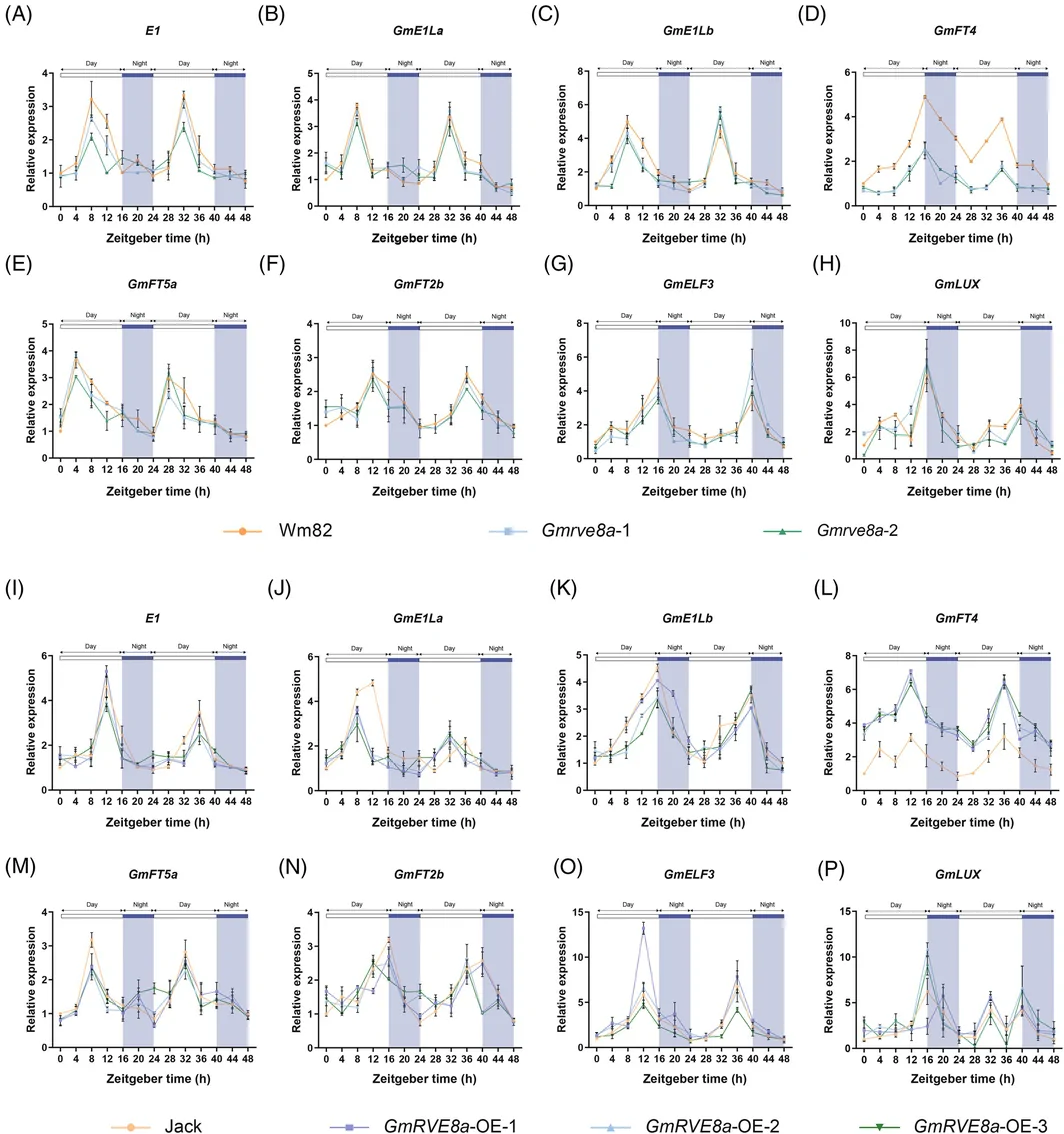
Diurnal expression patterns of flowering‐ and circadian‐related genes under long‐day (LD; 16 h light/8 h dark) conditions. (A–H) Relative expression levels of *E1*, *GmE1La*, *GmE1Lb*, *GmFT4*, *GmFT5a*, *GmFT2b*, *GmELF3*, and *GmLUX* in Wm82 and *Gmrve8a*‐mutant lines during a 48‐h time course under LD conditions. Wm82 is denoted by orange circles, *Gmrve8a*‐1 by blue squares, and *Gmrve8a*‐2 by green triangles. Wm82, Williams 82. (I–P) Relative expression levels of the same genes in Jack and *GmRVE8a*‐overexpression (*GmRVE8a*‐OE) lines during a 48‐h time course under LD conditions. Jack is denoted by orange circles, *GmRVE8a*‐OE‐1 by purple‐blue squares, *GmRVE8a*‐OE‐2 by light blue triangles, and *GmRVE8a*‐OE‐3 by green triangles. White and shaded areas indicate day and night periods, respectively. Zeitgeber time (ZT) is shown in hours. Data are presented as mean ± standard deviation of three independent biological replicates.

## DISCUSSION

### *GmRVE8a* negatively modulates flowering time and maturity in soybean

Flowering time and maturity are core agronomic traits that determine the ecological adaptability, yield formation, and chemical quality of soybean (Wang et al., 2024). Dissecting the molecular regulatory pathways controlling soybean flowering not only provides theoretical guidance for molecular breeding and optimized cultivation strategies but also has important implications for improving soybean productivity, promoting sustainable agricultural development, and ensuring food security.

Flowering and maturity in soybean are tightly regulated by the endogenous circadian clock and photoperiodic signaling pathways (Mao et al., 2017). Numerous studies have identified multiple key circadian clock genes involved in the regulation of soybean flowering and maturation, most of which act as negative regulators, including *GmPRR3a*, *GmPRR3b*, and *E2*, while only a few members mediate floral transition as positive regulators, such as *GmRVE1*, *GmELF3*, and *GmLUX* (Wu et al., 2023). Despite these advances, the circadian clock‐mediated photoperiodic flowering regulatory network in soybean still needs to be further improved, and the functions and molecular mechanisms of related gene family members have not been fully elucidated.

In this study, through GWAS, DL, haplotype analysis, CRISPR‐Cas9‐mediated functional validation, transgenic overexpression‐mediated functional validation, subcellular localization and expression pattern analysis, we suggest that *GmRVE8a* functions as a previously uncharacterized repressor of flowering and maturation in soybean. Comparison with characterized soybean *RVE* members helps define the role of *GmRVE8a*. *GmRVE1* promotes flowering through the *E1*‐*GmFT2a*/*GmFT5a* module, whereas *GmRVE5* mainly regulates hypocotyl elongation. In contrast to *GmRVE5*, *GmRVE8a* shows rhythmic expression and pronounced photoperiod‐dependent effects on flowering and maturity, indicating a closer functional relationship with *GmRVE1*. Given that *GmRVE1* functions downstream of *Tof11*/*Tof12* and upstream of *E1*, *GmRVE8a* may act at a comparable regulatory layer linking circadian cues with photoperiodic flowering and maturity control. Thus, *GmRVE8a* represents a distinct *RVE* regulator that is functionally related to *GmRVE1* but exerts an opposite effect on flowering time. Intriguingly, the *FT* gene family also exhibits similar functional differentiation. For instance, *GmFT4*, *GmFT1a*, and *GmFT1b* repress soybean flowering and maturation, whereas *GmFT2a* and *GmFT5a* function as positive regulators (Lee et al., 2021). This raises the possibility of specific regulatory relationships between members of the *RVE* and *FT* gene families. qRT‐PCR assays showed that *GmRVE8a* may delay flowering and maturity by promoting the expression of the floral repressor *GmFT4* and is unlikely to act by directly regulating *E1* expression. This finding not only enriches our understanding of the molecular regulatory network governing photoperiod‐ and circadian rhythm‐mediated flowering time in soybean but also provides a new research perspective and candidate gene for dissecting the molecular mechanisms of flowering time regulation in legume crops.

### *GmRVE8a* offers a promising molecular target for optimizing soybean adaptation across diverse geographical regions

Regional adaptability represents a core bottleneck restricting large‐scale cultivation and yield stability in soybean. Significant variations in photoperiod and temperature conditions across latitudes, altitudes, and climatic zones require soybean cultivars to precisely perceive and respond to these environmental signals to successfully complete their growth and developmental cycles and achieve high yields in target regions (Vollmann & Škrabišová, 2023). Traditional breeding approaches for improving soybean regional adaptability are often limited by long breeding cycles, low selection efficiency, and the difficulty of accurately selecting for complex polygenic traits. “Potalaization” has recently been proposed as a modern breeding strategy that integrates multiple molecular tools into elite, broadly adaptable cultivars to develop soybean varieties with wide regional adaptability (Qi et al., 2021).

In the present study, targeted mutagenesis of *GmRVE8a* in Wm82 via CRISPR‐Cas9 generated *Gmrve8a*‐mutant lines that exhibited significantly earlier maturity under NLD conditions. This early‐maturity phenotype may facilitate adaptation to high‐latitude environments and help reduce yield losses caused by early frost events. Conversely, *GmRVE8a‐*OE lines exhibited markedly delayed flowering and maturity, which may be favorable in low‐latitude regions, as the extended vegetative growth phase can enhance yield potential. These findings suggest that fine‐tuning *GmRVE8a* activity in different soybean genetic backgrounds could provide a demand‐oriented strategy for optimizing flowering and maturity according to regional production requirements. Collectively, this molecular target‐based precision breeding strategy can markedly shorten the breeding cycle and improve breeding efficiency, opening new avenues for developing soybean cultivars with broad adaptability, high yield, and stable production. Although our study supports *GmRVE8a* functions as a previously uncharacterized repressor of flowering and maturation in soybean, and suggests that it may delay reproductive transition by promoting the expression of the floral repressor *GmFT4* and is unlikely to act by directly regulating *E1* expression, several limitations remain. First, although *GmRVE8a* is located within the candidate interval of the identified QTL and shows strong functional relevance to flowering and maturity, allele‐specific functional validation or genome‐editing experiments will be required to establish whether *GmRVE8a* is the causal gene underlying this QTL. Second, based on qRT‐PCR evidence, we were able to show that *GmFT4* may act downstream of *GmRVE8a*; however, these expression data alone cannot distinguish whether *GmRVE8a* regulates *GmFT4* directly or indirectly. To provide additional sequence‐level context for this regulatory relationship, we examined the 2‐kb upstream regulatory region of *GmFT4*, relative to the translation start site, for Evening Element (EE) motifs, which are commonly associated with binding by RVE‐type transcription factors. Two EE‐like core motifs with the sequence AAATATCT were identified in the *GmFT4* upstream region, located at −714 to −707 bp and −250 to −243 bp relative to the ATG start codon (Figure S10). The presence of these EE‐like motifs suggests that the *GmFT4* promoter contains potential cis‐regulatory elements that could support regulation by *GmRVE8a*. Nevertheless, motif occurrence alone does not demonstrate direct transcription factor binding or transcriptional activation. We have not yet validated the direct transcriptional targets of *GmRVE8a* via chromatin immunoprecipitation sequencing (ChIP‐seq) and corresponding *in vitro* and *in vivo* binding assays. Therefore, the precise molecular mechanism by which *GmRVE8a* regulates *GmFT4* expression remains to be fully elucidated.

## MATERIALS AND METHODS

### Population construction, growth conditions, and phenotypic analysis

The experimental materials consisted of a natural population of 257 soybean accessions collected worldwide, which were classified into 14 continuously distributed maturity groups (MG0000‐MGX). This population included 214 cultivated varieties, 33 landraces, and 10 wild soybean accessions. Soybean seeds were obtained from the Key Laboratory of Soybean Biology, Ministry of Agriculture and Rural Affairs, Institute of Crop Sciences (ICS), Chinese Academy of Agricultural Sciences (CAAS), Beijing, China. To confirm the maturity dates, the natural population was planted in CAAS Shunyi experimental base (Beijing) (40°23′ N, 116°57′ E) in both 2020 and 2021. For the determination of flowering and maturity stages, the Jack, Wm82, *GmRVE8a* transgenic lines, and *Gmrve8a* mutants were cultivated under the following conditions: open‐field conditions from June to October in Beijing and Xinxiang (35°10′ N, 113°48′ E); controlled conditions in a growth chamber with SD and LD. Each unique location–year combination was treated as a separate testing environment, yielding six environments: BJ2020, BJ2021, BJ2023, BJ2025, XX2023, and XX2025. Based on the standard classification of soybean developmental stages (Fehr & Caviness, 1977), 12 plants were randomly selected and tagged to record three key developmental stages: emergence (VE, the date when cotyledons emerged above the soil surface), beginning bloom (R1, denoting the date of first flowering on the main stem), and physiological maturity (R7, denoting the date when at least one normal main‐stem pod reached physiological maturity).

### Primer sequences and gene accession information

The sequences of all primers employed in this study are summarized in Table S5. The corresponding gene sequences were downloaded from the Phytozome database based on the *Glycine max* Wm82.a2.v1 genome assembly, using the accession numbers listed below: *GmRVE8a* (*Glyma.03G177300*), *GmRVE8b* (*Glyma.13G136300*), *E1* (*Glyma.06G207800*), *GmLUX* (*Glyma.12G060200*), *GmFT5a* (*Glyma.16G044100*), *GmFT2b* (*Glyma.16G151000*), *GmFT4* (*Glyma.08G363100*), *GmE1La* (*Glyma.04G156400*), *GmE1Lb* (*Glyma.04G143300*), *GmELF3* (*Glyma.04G050200*), and *GmActin* (*Glyma.18G290800*).

### DNA extraction, sequencing, and quality control

Total genomic DNA of each sample was extracted using the Vazyme Plant Genomic DNA Extraction Kit. After extraction, DNA purity was determined with a NanoDrop 2000 ultraviolet spectrophotometer (Thermo Fisher Scientific, Waltham, MA, USA). Samples were screened for quality based on the criteria that the A260/A280 ratio ranged from 1.8 to 2.0 and the A260/A230 ratio was no less than 2.0. Meanwhile, 1.0% agarose gel electrophoresis was performed to verify DNA integrity, and high‐quality DNA with clear bands and no obvious degradation was selected for subsequent experiments. Following fragmentation of high‐quality DNA samples, the Vazyme Library Construction Kit was used to complete library preparation steps, including end repair, A‐tailing, adapter ligation, and PCR amplification, resulting in the construction of sequencing libraries with an insert size of approximately 350 bp. After passing library quality control, paired‐end whole‐genome resequencing was conducted on the Illumina NovaSeq sequencing platform with a read length of 150 bp, generating raw sequencing data (Raw Reads). Raw Reads were filtered using Trimmomatic software (Bolger et al., 2014) to remove adapter‐containing sequences, low‐quality reads (Q30 <80%), and sequences with an N content of ≥10%, yielding high‐quality clean reads (Clean Reads). The BWA‐MEM algorithm (Li & Durbin, 2009) was employed to map Clean Reads to the soybean reference genome (*Glycine max* Wm82.a2.v1) under default parameters, achieving accurate sequence alignment. Sequence assembly, variant calling, and annotation of the aligned sequence data were collaboratively accomplished using SAMtools software (Li et al., 2009), BCFtools software (Li, 2011), and SnpEff software (Cingolani et al., 2012). To obtain high‐quality SNP loci, VCFtools software (Danecek et al., 2011) was used for further stringent filtering of variant data with the following parameters: maximum missing rate (max‐missing) ≤0.2 and minor allele frequency (MAF) ≥0.01. The filtered variant data were subjected to sequencing file format conversion using PLINK software (Purcell et al., 2007) and TASSEL software (Bradbury et al., 2007). Meanwhile, InDel sites were excluded, ultimately obtaining a high‐quality SNP dataset for subsequent analyses.

### Phylogenetic structure and linkage disequilibrium analysis

Based on the filtered high‐quality SNPs dataset, population phylogenetic structure analysis was performed to reveal the genetic relationships among germplasm materials. PCA was conducted using the Princomp() function (Price et al., 2006). The visualization of PCA results was carried out with the “Scatterplot3d” package, and the first three principal components were extracted to present the population clustering characteristics (Ligges & Mächler, 2003). A population phylogenetic tree was constructed using SNPhylo software, employing the Neighbor‐Joining (NJ) method with 1000 Bootstrap replicates to assess the reliability of node support (Lee et al., 2014). A heatmap of the population kinship matrix was plotted using the “ComplexHeatmap” package to intuitively display the genetic relatedness among materials (Gu et al., 2016). The SNP distribution density map across the whole genome was generated using the “CMplot” package to clarify the distribution characteristics of SNP loci (https://github.com/YinLiLin/R-CMplot). Using the same high‐quality SNPs dataset, the linkage degree between SNPs was calculated by PopLDdecay software. Linkage disequilibrium decay was estimated based on the *r* ^2^ values between pairs of SNPs, which were averaged in 1‐kb windows with a maximum distance of 1 Mb (Zhang et al., 2019). The threshold for linkage disequilibrium was set as the point where the *r* ^2^ value decays to half of its maximum, so as to determine the linkage disequilibrium decay distance of the population (Huang et al., 2010).

### GWAS of growth period traits

GWAS analyses of maturity‐related traits were conducted in GAPIT v3 (http://zzlab.net/GAPIT) using a filtered set of 2 757 937 high‐quality SNPs (Wang & Zhang, 2021) with the Bayesian‐information and Linkage‐disequilibrium Iteratively Nested Keyway (BLINK). With the number of SNPs analyzed (*n* = 2 757 937), the threshold for significance was estimated to be approximately *P* = 1 × 10^−7^ by the Bonferroni correction method. Manhattan and quantile‐quantile (QQ) plots were constructed with the qqman R package (Turner, 2014) to visualize the distribution of phenotypic values for each SNP and evaluate the association performance of the BLINK.

### Identification of candidate genes using DL

The deep learning tool DeepGOPlus (Kulmanov & Hoehndorf, 2020) was employed, with a prediction threshold set at 0.3. Under this threshold condition, molecular‐level features of proteins were accurately mined, screened, and extracted from amino acid sequences. The entire process relied on the DL capability of this algorithm to ensure the accuracy and specificity of feature extraction.

### Haplotype and genetic diversity analysis

A variety of bioinformatics tools were adopted to perform haplotype and genetic diversity analysis for dissecting the genetic variation characteristics of the target gene and population. Python scripts were used to conduct haplotype analysis on the SNP/InDel loci within the exons of *GmRVE8a*. PopART v1.7 software was utilized to plot geographical distribution maps to reflect the spatial distribution characteristics of different haplotypes.

### Sample collection, RNA extraction, and qRT‐PCR analysis

To characterize the tissue‐specific expression profile of *GmRVE8a*, various tissues, including roots, hypocotyls, cotyledons, stems, true leaves, trifoliolate leaves, shoot apical meristems, flowers, pods, and seeds, were collected from soybean plants. *GmRVE8b* was also examined using the same qRT‐PCR method. For rhythmic expression analysis, Jack, Wm82, *GmRVE8a* transgenic lines, and *Gmrve8a* mutants were cultivated in growth chambers under SD and LD photoperiods for 3 weeks, after which fully expanded trifoliolate leaves with uncurled margins were harvested at 4‐h intervals throughout a 48‐h diurnal cycle. The transcript levels of *GmRVE8a* and flowering‐related genes, including *E1*, *GmE1La*, *GmE1Lb*, *GmFT4*, *GmFT5a*, *GmFT2b*, *GmELF3*, and *GmLUX*, were analyzed. Total RNA extraction and cDNA synthesis for all collected samples were performed using the RNA Extraction and Reverse Transcription Kit (Vazyme, Nanjing, China). qRT‐PCR amplification and detection were conducted on a 96‐well optical plate using the SYBR® Premix Ex Taq™ II Kit, with the mRNA expression level of soybean *GmActin* gene serving as the internal reference to calibrate the detection results of all samples.

### Vector construction and genetic transformation

For overexpression analysis, the CDS of the *GmRVE8a* ^*H1*^ was amplified from a soybean accession carrying *GmRVE8a* ^*H1*^ using KOD‐One DNA Polymerase (TOYOBO Life Science, Osaka, Japan), with the primers listed in Table S5. Following electrophoretic separation on a 1.0% agarose gel, the PCR amplicons were excised from the gel and purified using the V‐Elute Gel Mini Purification Kit (Zomanbio). The linearized PTF101.1‐GFP vector was subjected to directional recombination with *GmRVE8a*, and the resulting construct was named *GmRVE8a*‐OE. Based on the genome sequence, appropriate single guide RNA (sgRNA) target sequences were screened using the online tool CRISPR‐P v2.0 (http://crispr.hzau.edu.cn/cgi-bin/CRISPR2/CRISPR) (Liu et al., 2017). Primers for the sgRNA target sequence, including forward and reverse complementary sequences, were designed and synthesized. All constructed vectors were transformed into *Agrobacterium tumefaciens* strain EH105, followed by introduction into soybean cultivar Williams 82 (Chen et al., 2018).

### Subcellular localization

*Agrobacterium tumefaciens* suspension containing the *GmRVE8a*‐OE plasmid (OD₆₀₀ value of approximately 0.2) was infiltrated into the leaves of 6 to 8 weeks *Nicotiana benthamiana* plants using a syringe without a needle for transient expression (Yang et al., 2000). After incubation in the dark at 22°C for 48 h, the leaves were observed under a laser confocal scanning microscope (ZEISS LSM 700, Jena, Germany).

### Statistical analysis

For phenotypic evaluation, 12 plants were counted per measurement. For qRT‐PCR expression analysis, each tissue sample was harvested from at least three individual plants, with a minimum of three technical replicates for each sample. All phenotypic and gene expression data were statistically analyzed by one‐way analysis of variance (ANOVA), followed by Duncan's multiple range test for multiple comparisons using SPSS software (v.23, IBM). Significant differences were denoted by different lowercase letters in the corresponding figures, and detailed statistical results are described in the figure legends.

## AUTHOR CONTRIBUTIONS

SS performed the experiments and drafted the manuscript. TW was responsible for natural population sampling. BJ curated and provided the sequencing resources. HJ, XS, MY, XY, PW, LJ, and YS carried out the phenotypic analyses. SY, CQ, ZQ, and SS contributed to manuscript review and editorial refinement. BS, TH, and HL conceptualized the study, established the research framework, and participated in manuscript revision. All authors read and approved the final manuscript.

## CONFLICT OF INTEREST

The authors declare that they have no conflicts of interest in relation to the content of this manuscript.

## Supporting information


**Figure S1.** Principal component analysis (PCA) of 257 soybean germplasm accessions. HR, accessions from the Huang–Huai–Hai Summer Planting Region; NR, accessions from the Northern Spring Planting Region; SR, accessions from the Southern Multiple Cropping Region.

**Figure S2.** Genome‐wide linkage disequilibrium decay plot. Genome‐wide linkage disequilibrium decay estimated as the squared correlation coefficient (*r* ^2^) between SNP pairs against physical distance.

**Figure S3.** Frequency distribution of days from emergence to physiological maturity in the 257 soybean germplasm accessions. Red curve, fitted normal distribution. VE, emergence; R7, physiological maturity; VE‐R7, days from VE to R7; d, day.

**Figure S4.** Quantile–quantile (QQ) plot for genome‐wide association analyses of soybean maturity. The red dashed line indicates the expected distribution under the null hypothesis.

**Figure S5.** Tissue‐specific expression pattern of *GmRVE8a* in diverse organs.

**Figure S6.** Alignment of amino acid sequences of *GmRVE8a* haplotypes and its homologs. (a) In silico expression profile across soybean tissues. (b) Transcript levels of *GmRVE8a* in different organs. Data are presented as mean ± standard deviation of three independent biological replicates. Different lowercase letters indicate statistically significant differences at the *p* < 0.05 level as determined by Duncan's multiple range test.

**Figure S7.** Homozygous targeted mutagenesis of *GmRVE8a* mediated by CRISPR‐Cas9 in *Gmrve8a*‐mutant lines. Schematic representation of the *GmRVE8a* gene structure and the CRISPR‐Cas9 target site in the exon. Exons and introns are indicated. ATG and TGA denote the translation start and stop codons, respectively. Two independent homozygous mutant alleles were identified in the Williams 82 background, including a 4‐bp deletion and a 5‐bp deletion. Red arrows indicate the mutation sites. Wm82, Williams 82.

**Figure S8.** Phenotypic characterization of *Gmrve8a*‐mutant and *GmRVE8a*‐overexpression (*GmRVE8a*‐OE) transgenic soybean lines under short‐day (SD; 12 h light/12 h dark) and long‐day (LD; 16 h light/8 h dark) conditions. (a, c) Flowering time (days from VE to R1) of *Gmrve8a* mutant lines across SD and LD conditions. VE, emergence; R1, beginning bloom. Wm82, Williams 82. (b, d) Maturity (days from VE to R7) of *Gmrve8a* mutant lines across SD and LD conditions. R7, physiological maturity. (e, g) Flowering time (days from VE to R1) of *GmRVE8a*‐*OE* lines across SD and LD conditions. (f, h) Maturity (days from VE to R7) of *GmRVE8a*‐*OE* lines across SD and LD conditions. *n* = 12 plants. d, day. Different lowercase letters indicate statistically significant differences at the *p* < 0.05 level as determined by Duncan's multiple range test.

**Figure S9.** Diurnal expression patterns of flowering‐ and circadian‐related genes under short‐day (SD) conditions. (a–h) Relative expression levels of *E1*, *GmE1La*, *GmE1Lb*, *GmFT4*, *GmFT5a*, *GmFT2b*, *GmELF3*, and *GmLUX* in Wm82 and *Gmrve8a* mutant lines over a 48‐h time course under SD conditions. Wm82 is denoted by orange circles, *Gmrve8a*‐1 by blue squares, and *Gmrve8a*‐2 by green triangles. (i–p) Relative expression levels of the same genes in Jack and *GmRVE8a*‐overexpression (*GmRVE8a*‐OE) lines over a 48‐h time course under SD conditions. Jack is denoted by light orange circles, *GmRVE8a*‐OE‐1 by purple‐blue squares, *GmRVE8a*‐OE‐2 by light blue triangles, and *GmRVE8a*‐OE‐3 by green triangles. White and shaded areas indicate day and night periods, respectively. Zeitgeber time (ZT) is shown in hours. Data are presented as mean ± standard deviation of three independent biological replicates.

**Figure S10.** Schematic representation of the 2‐kb upstream regulatory region of *GmFT4* showing the distribution of Evening Element (EE)‐like motifs. The translation start codon ATG is designated as position 0. Two EE‐like core motifs with the sequence AAATATCT were identified at positions −714 to −707 bp and ‐250 to −243 bp relative to the ATG start codon. Blue square, EE‐like 1; green square, EE‐like 2.

**Table S1.** Sequencing summary of 257 re‐sequenced soybean accessions. Variety, designation name assigned to each soybean germplasm accession; Raw reads, total number of raw sequencing reads generated from the sequencing library; Clean reads, total number of high‐quality reads retained after quality filtering; Q30 (%), percentage of bases with a Phred quality score ≥30, representing base‐level sequencing accuracy; Coverage (%), proportion of the reference genome covered by at least one uniquely mapped clean read.

**Table S2.** Genomic positions of significant GWAS SNPs and corresponding candidate genes in 257 soybean accessions. SNP identifiers are presented in the format “chromosome‐position”. Chromosome indicates the soybean chromosome on which the SNP is located, and Position refers to the physical coordinate of the SNP on the reference genome. *p* value represents the significance level of the marker‐trait association from the GWAS analysis. Candidate gene indicates the annotated gene located at or nearest to the associated SNP, based on the reference genome annotation. “Unknown” indicates that no previously annotated or confidently assigned candidate gene was identified for that SNP locus.

**Table S3.** Predicted genes in mapping region (38 989‐kb to 39 087‐kb) according to *Glycine max* Wm82.a2.v1 reference genome. Gene ID indicates the annotated soybean locus identifier. Function represents the predicted biological function or annotation assigned in the reference genome. These genes were considered positional candidates within the target mapping region.

**Table S4.** Gene ontology (GO) enrichment scores for biological process (BP) in the candidate genomic region linked to soybean maturity, calculated using the deep learning‐based DeepGOPlus model. The identified GO terms were classified into three major categories: Cellular Component, Molecular Function, and Biological Process.

**Table S5.** Primers used in this study.

## Data Availability

All whole genome sequencing data in this study are available from NCBI under accession number PRJNA1114037 (https://www.ncbi.nlm.nih.gov/bioproject/PRJNA1114037). The *Glycine max* Wm82.a2.v1 reference genome was obtained from Phytozome (https://phytozome-next.jgi.doe.gov/info/Gmax_Wm82_a2_v1). All other data supporting the findings of this study are provided in the article and its Supplementary Information files.
