# Supplementary material for: GmRVE8a, a circadian clock‐associated gene, negatively regulates flowering and maturity in soybean
Source: Plant J. 2026 Nov 4;128(3):e71205. doi: 10.1111/tpj.71205 (PMC13732551; doi:10.1111/tpj.71205)
Supplement: Supplementary file 1 [file TPJ-128-0-s001.pdf]

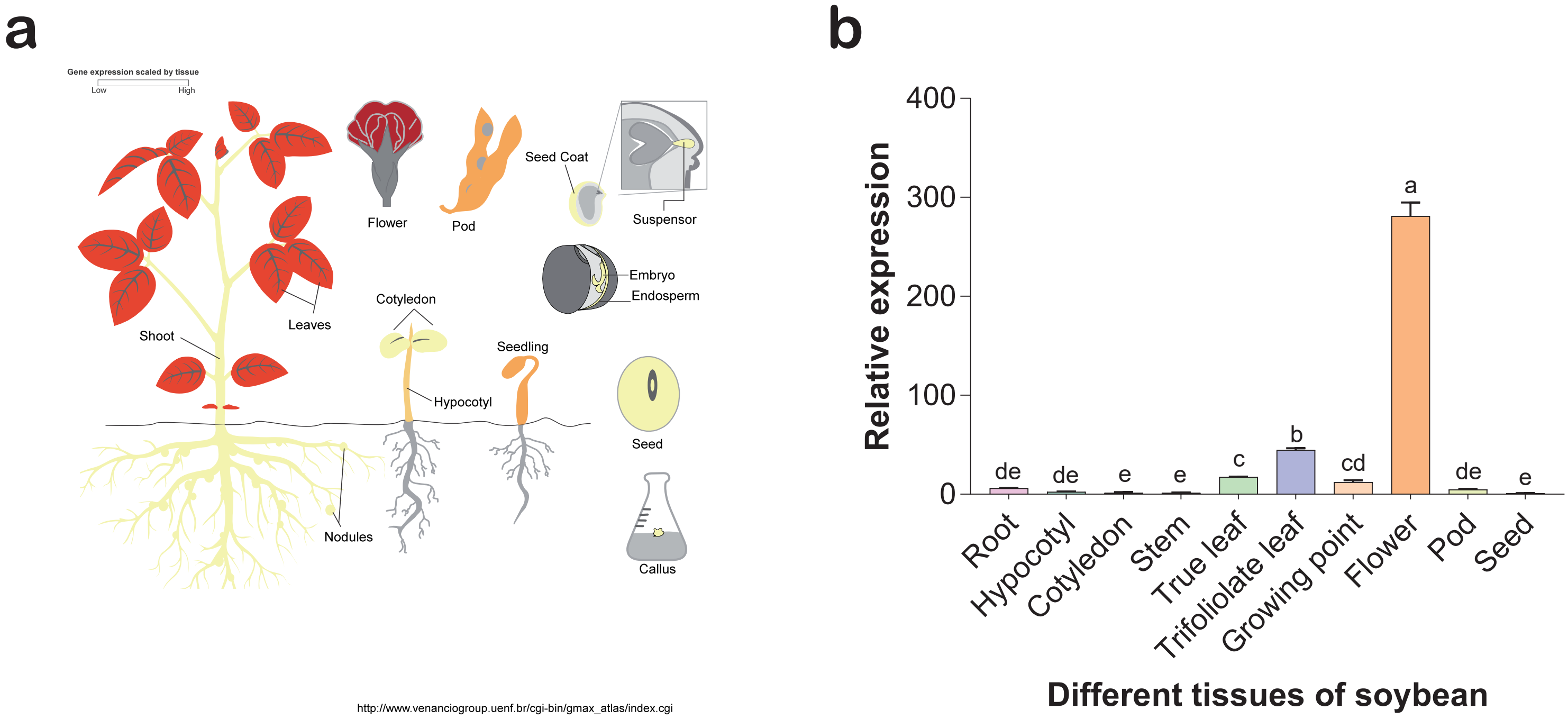
a
Gene expression scaled by tissue
Low
High
Flower
Pod
Seed Coat
Suspensor
Embryo
Endosperm
Leaves
Shoot
Cotyledon
Seedling
Hypocotyl
Seed
Nodules
Callus
http://www.venanciogroup.uenf.br/cgi-bin/gmax_atlas/index.cgi
b
Relative expression
400
300
200
100
0
de
de
e
e
c
b
cd
a
de
e
Root
Hypocotyl
Cotyledon
Stem
True leaf
Trifoliolate leaf
Growing point
Flower
Pod
Seed
Different tissues of soybean
