# Supplementary material for: GmRVE8a, a circadian clock‐associated gene, negatively regulates flowering and maturity in soybean
Source: Plant J. 2026 Nov 4;128(3):e71205. doi: 10.1111/tpj.71205 (PMC13732551; doi:10.1111/tpj.71205)
Supplement: Supplementary file 2 [file TPJ-128-0-s002.pdf]

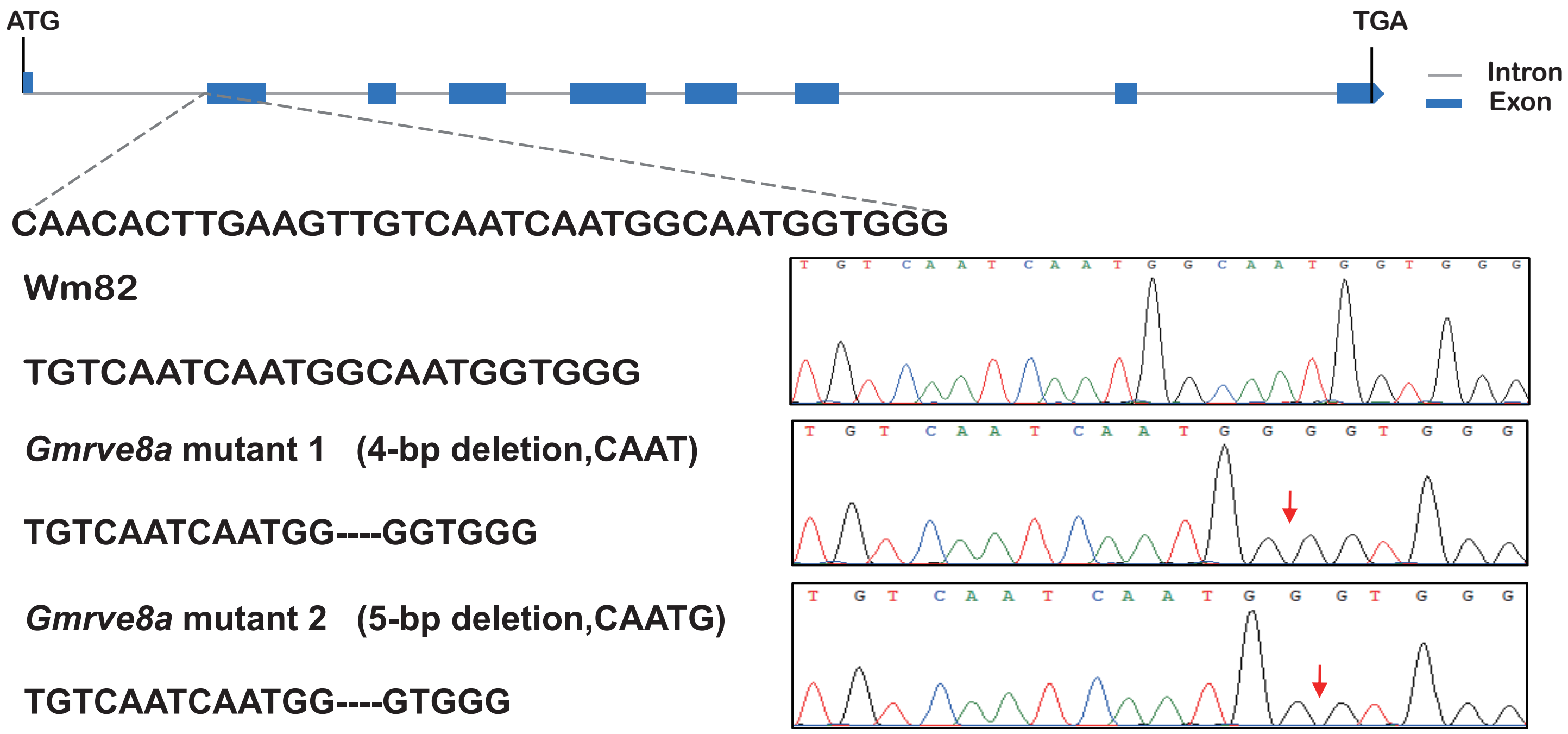
ATG
TGA
Intron
Exon
CAACACTTGAAGTTGTCAATCAATGGCAATGGTGGG
Wm82
TGTCAATCAATGGCAATGGTGGG
Gmrve8a mutant 1 (4-bp deletion,CAAT)
TGTCAATCAATGG----GGTGGG
Gmrve8a mutant 2 (5-bp deletion,CAATG)
TGTCAATCAATGG----GTGGG
