# Supplementary material for: GmRVE8a, a circadian clock‐associated gene, negatively regulates flowering and maturity in soybean
Source: Plant J. 2026 Nov 4;128(3):e71205. doi: 10.1111/tpj.71205 (PMC13732551; doi:10.1111/tpj.71205)
Supplement: Supplementary file 3 [file TPJ-128-0-s004.pdf]

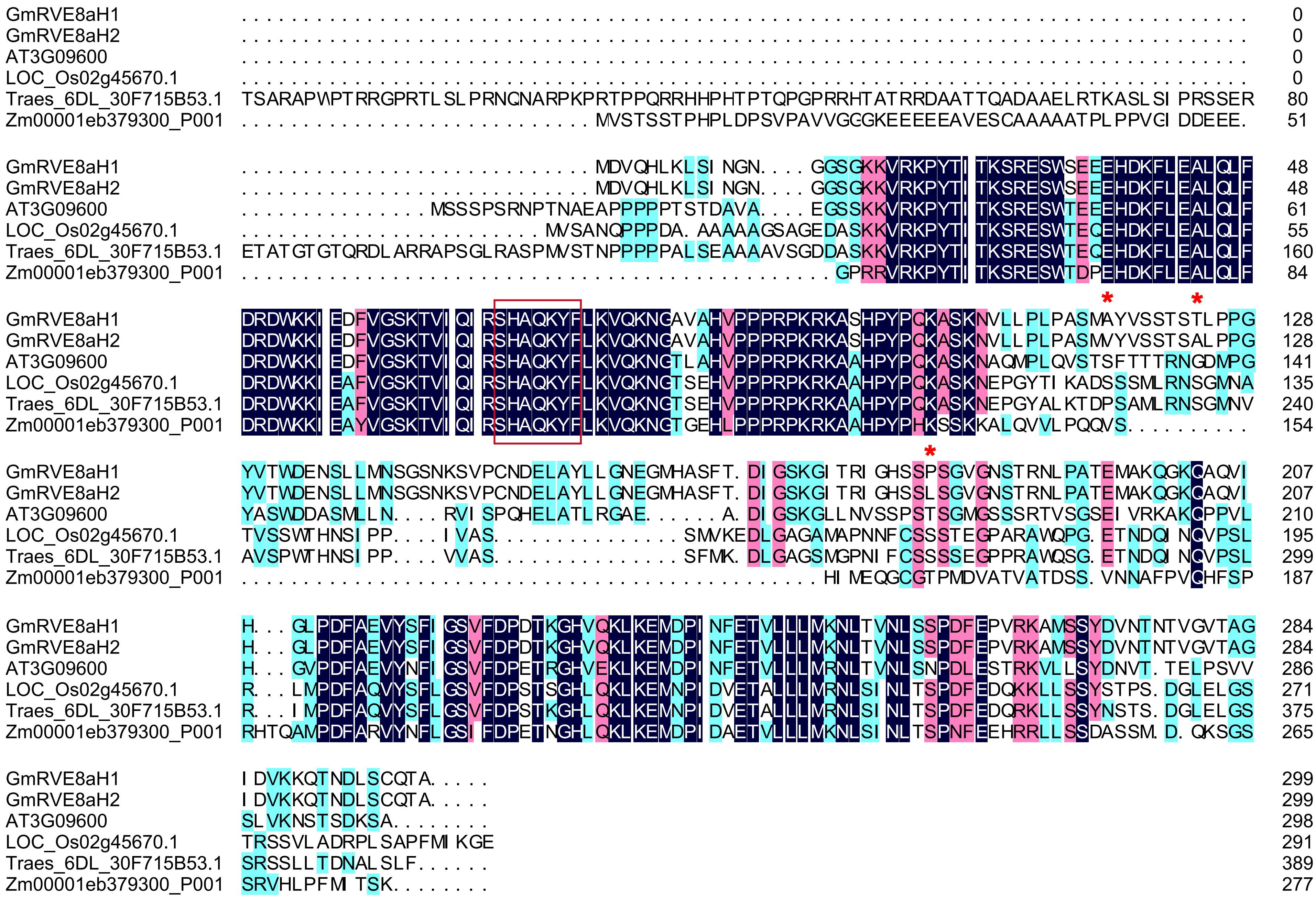

GmRVE8aH1 0
GmRVE8aH2 0
AT3G09600 0
LOC_Os02g45670.1 0
Traes_6DL_30F715B53.1 TSARAPWPTRRGPRTLSLPRNCNARPKPRTPPQRRHHPHTPTQPGPRRHTATRRDAATTQADAAELRTKASLSIPRSSER 80
Zm00001eb379300_P001 ..........................MVSTSSTPHPLDPSVPAVVGGGKEEEEEAVESCAAAAATPLPPVGIDDEEE. 51
GmRVE8aH1 ..........................MDVQHLKLSINGN....GGSGKKVRKPYTITKSRESWSEEEHDKFLEALQLF 48
GmRVE8aH2 ..........................MDVQHLKLSINGN....GGSGKKVRKPYTITKSRESWSEEEHDKFLEALQLF 48
AT3G09600 ...............MSSSPSRNPTNAEAPPPPPTSTDAVA....EGSSKKVRKPYTITKSRESWTEEEHDKFLEALQLF 61
LOC_Os02g45670.1 ....MVSANQPPPDA.AAAAAGSAGEDASKKVRKPYTITKSRESWTEQEHDKFLEALQLF 55
Traes_6DL_30F715B53.1 ETATGTGTQRDLARRAPSGLRASPMVSTNPPPPPALSEAAAAVSGDDASKKVRKPYTITKSRESWTEQEHDKFLEALQLF 160
Zm00001eb379300_P001 ....GPRRVRKPYTITKSRESWTDPEHDKFLEALQLF 84
GmRVE8aH1 DRDWKKIEDFVGSKTVIQIRSHAQKYFLKVQKNGAVAHVPPPRPKRKASHPYPCKASKNVLLPLPASMAYVSSTSTLPPG 128
GmRVE8aH2 DRDWKKIEDFVGSKTVIQIRSHAQKYFLKVQKNGAVAHVPPPRPKRKASHPYPCKASKNVLLPLPASMVYVSSTSALPPG 128
AT3G09600 DRDWKKIEDFVGSKTVIQIRSHAQKYFLKVQKNGTLAHVPPPRPKRKAAHPYPQKASKNAQMPLQVSTSFTTTRNGDMPG 141
LOC_Os02g45670.1 DRDWKKIEAFVGSKTVIQIRSHAQKYFLKVQKNGTSEHVPPPRPKRKAAHPYPCKASKNEPGYTIKADSSSMLRNSGMNA 135
Traes_6DL_30F715B53.1 DRDWKKIEAFVGSKTVIQIRSHAQKYFLKVQKNGTSEHVPPPRPKRKAAHPYPCKASKNEPGYALKTDPSAMLRNSGMNV 240
Zm00001eb379300_P001 DRDWKKIEAYVGSKTVIQIRSHAQKYFLKVQKNGTGEHLPPPRPKRKAAHPYPHKSSKKALQVVLPQQVS.......... 154
GmRVE8aH1 YVTWDENSLLMNSGSNKSVPCNDELAYLLGNEGMHASFT.DIGSKGITRIGHSSPSGVGNSTRNLPATEMAKQGKCAQVI 207
GmRVE8aH2 YVTWDENSLLMNSGSNKSVPCNDELAYLLGNEGMHASFT.DIGSKGITRIGHSSLSGVGNSTRNLPATEMAKQGKCAQVI 207
AT3G09600 YASWDDASMLLN....RVISPQHELATLRGAE......A.DIGSKGLLNVSSPSTSGMGSSSRTVSGSEIVRKAKCPPVL 210
LOC_Os02g45670.1 TVSSWTHNSIPP....IVAS...............SMVKEDLGAGAMAPNNFCSSSTEGPARAWQPG.ETNDQINCVPSL 195
Traes_6DL_30F715B53.1 AVSPWTHNSIPP....VVAS...............SFMK.DLGAGSMGPNIFCSSSSEGPPRAWQSG.ETNDQINCVPSL 299
Zm00001eb379300_P001 ....HIMEQGCGTPMDVATVATDSS.VNNAFPVQHFSP 187
GmRVE8aH1 H...GLPDFAEVYSFIGSVFDPDTKGHVQKLKEMDPINFETVLLLMKNLTVNLSSPDFEPVRKAMSSYDVNTNTVGVTAG 284
GmRVE8aH2 H...GLPDFAEVYSFIGSVFDPDTKGHVQKLKEMDPINFETVLLLMKNLTVNLSSPDFEPVRKAMSSYDVNTNTVGVTAG 284
AT3G09600 H...GVPDFAEVYNFIGSVFDPETRGHVEKLKEMDPINFETVLLLMRNLTVNLSNPDLESTRKVLLSYDNVT.TELPSVV 286
LOC_Os02g45670.1 R...LMPDFAQVYSFLGSVFDPSTSGHLQKLKEMNPIDVETALLLMRNLSINLTSPDFEDQKKLLSSYSTPS.DGLELGS 271
Traes_6DL_30F715B53.1 R...IMPDFAQVYSFLGSVFDPSTKGHLQKLKEMNPIDVETALLLMRNLSINLTSPDFEDQRKLLSSYNSTS.DGLELGS 375
Zm00001eb379300_P001 RHTQAMPDFARVYNFLGSIFDPETNGHLQKLKEMDPIDAETVLLLMKNLSINLTSPNFEEHRRLLSSDASSM.D.QKSGS 265
GmRVE8aH1 IDVKKQTNDLSCQTA..... 299
GmRVE8aH2 IDVKKQTNDLSCQTA..... 299
AT3G09600 SLVKNSTSDKSA........ 298
LOC_Os02g45670.1 TRSSVLADRPLSAPFMIKGE 291
Traes_6DL_30F715B53.1 SRSSLLTDNALSLF...... 389
Zm00001eb379300_P001 SRVHLPFMITSK........ 277
