# Supplementary material for: GmRVE8a, a circadian clock‐associated gene, negatively regulates flowering and maturity in soybean
Source: Plant J. 2026 Nov 4;128(3):e71205. doi: 10.1111/tpj.71205 (PMC13732551; doi:10.1111/tpj.71205)
Supplement: Supplementary file 4 [file TPJ-128-0-s005.pdf]

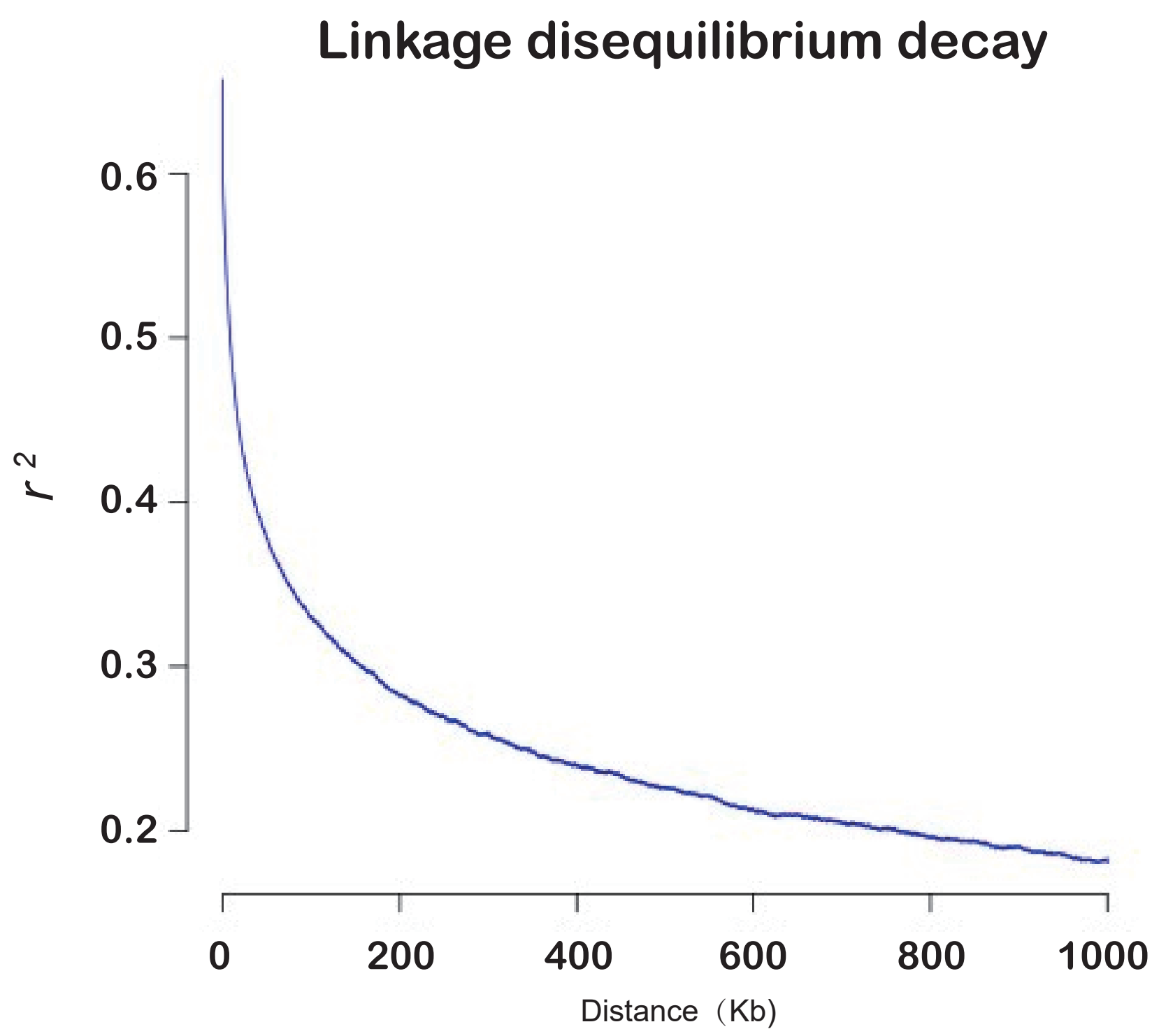
Linkage disequilibrium decay
0.6
0.5
0.4
0.3
0.2
r 2
0
200
400
600
800
1000
Distance（Kb)
