# Supplementary material for: GmRVE8a, a circadian clock‐associated gene, negatively regulates flowering and maturity in soybean
Source: Plant J. 2026 Nov 4;128(3):e71205. doi: 10.1111/tpj.71205 (PMC13732551; doi:10.1111/tpj.71205)
Supplement: Supplementary file 5 [file TPJ-128-0-s006.pdf]

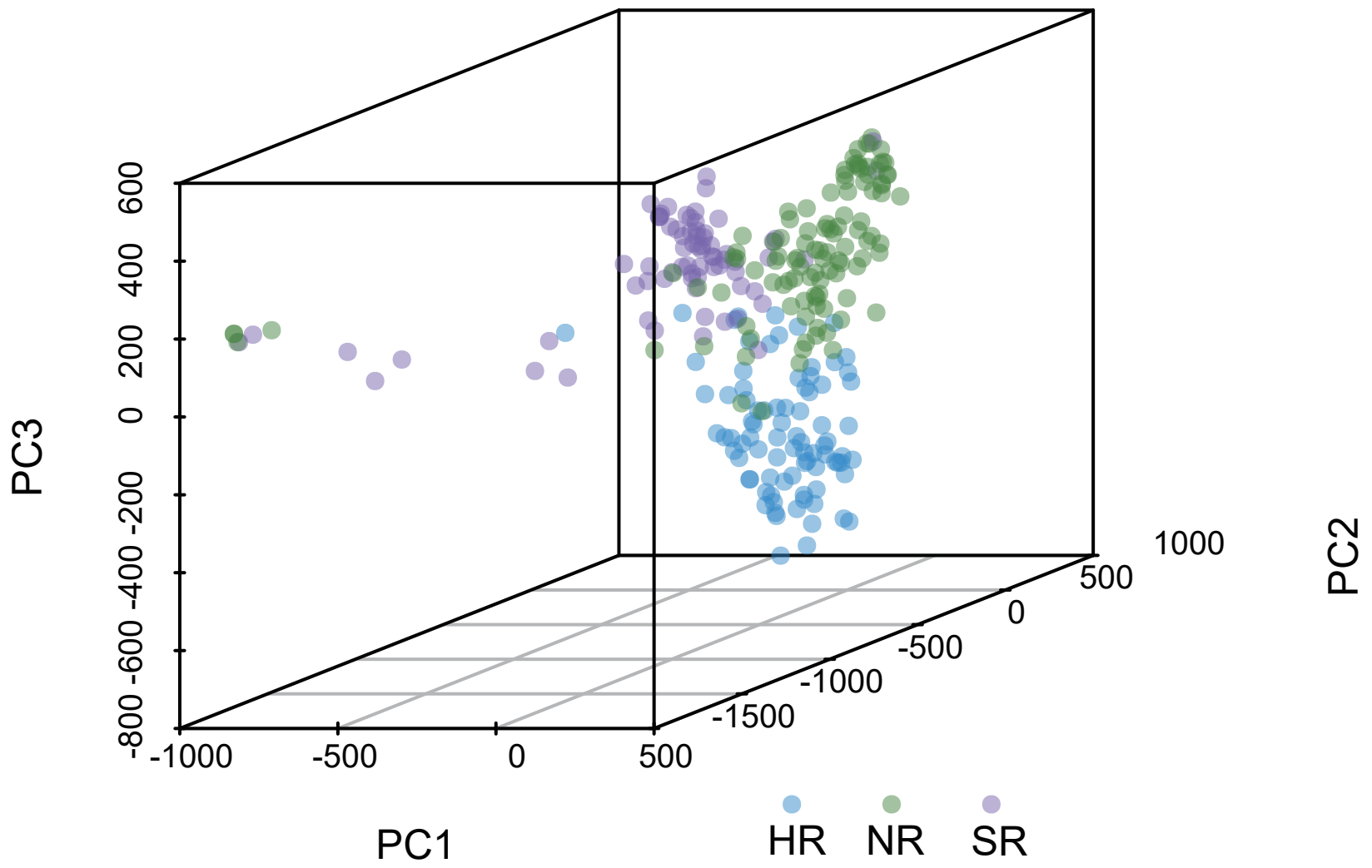
PC3
600
400
200
0
-200
-400
-600
-800
-1000
-500
0
500
PC1
PC2
1000
500
0
-500
-1000
-1500
HR
NR
SR
