# Supplementary material for: GmRVE8a, a circadian clock‐associated gene, negatively regulates flowering and maturity in soybean
Source: Plant J. 2026 Nov 4;128(3):e71205. doi: 10.1111/tpj.71205 (PMC13732551; doi:10.1111/tpj.71205)
Supplement: Supplementary file 6 [file TPJ-128-0-s007.pdf]

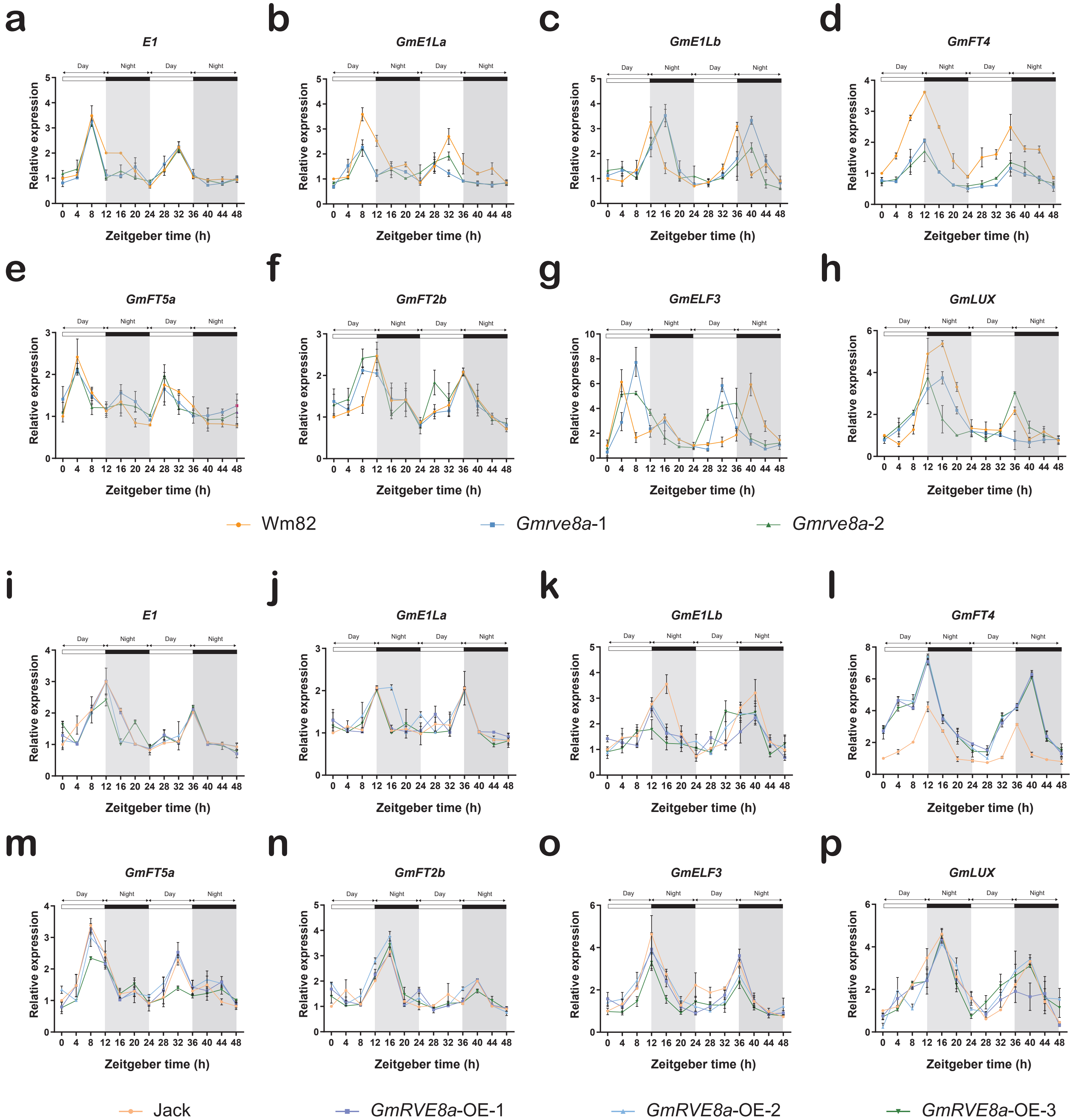

a
E1
b
GmE1La
c
GmE1Lb
d
GmFT4
e
GmFT5a
f
GmFT2b
g
GmELF3
h
GmLUX
Day
Night
Relative expression
Zeitgeber time (h)
0 4 8 12 16 20 24 28 32 36 40 44 48
Wm82
Gmrve8a-1
Gmrve8a-2
i
E1
j
GmE1La
k
GmE1Lb
l
GmFT4
m
GmFT5a
n
GmFT2b
o
GmELF3
p
GmLUX
Jack
GmRVE8a-OE-1
GmRVE8a-OE-2
GmRVE8a-OE-3
