# Supplementary material for: GmRVE8a, a circadian clock‐associated gene, negatively regulates flowering and maturity in soybean
Source: Plant J. 2026 Nov 4;128(3):e71205. doi: 10.1111/tpj.71205 (PMC13732551; doi:10.1111/tpj.71205)
Supplement: Supplementary file 7 [file TPJ-128-0-s008.pdf]

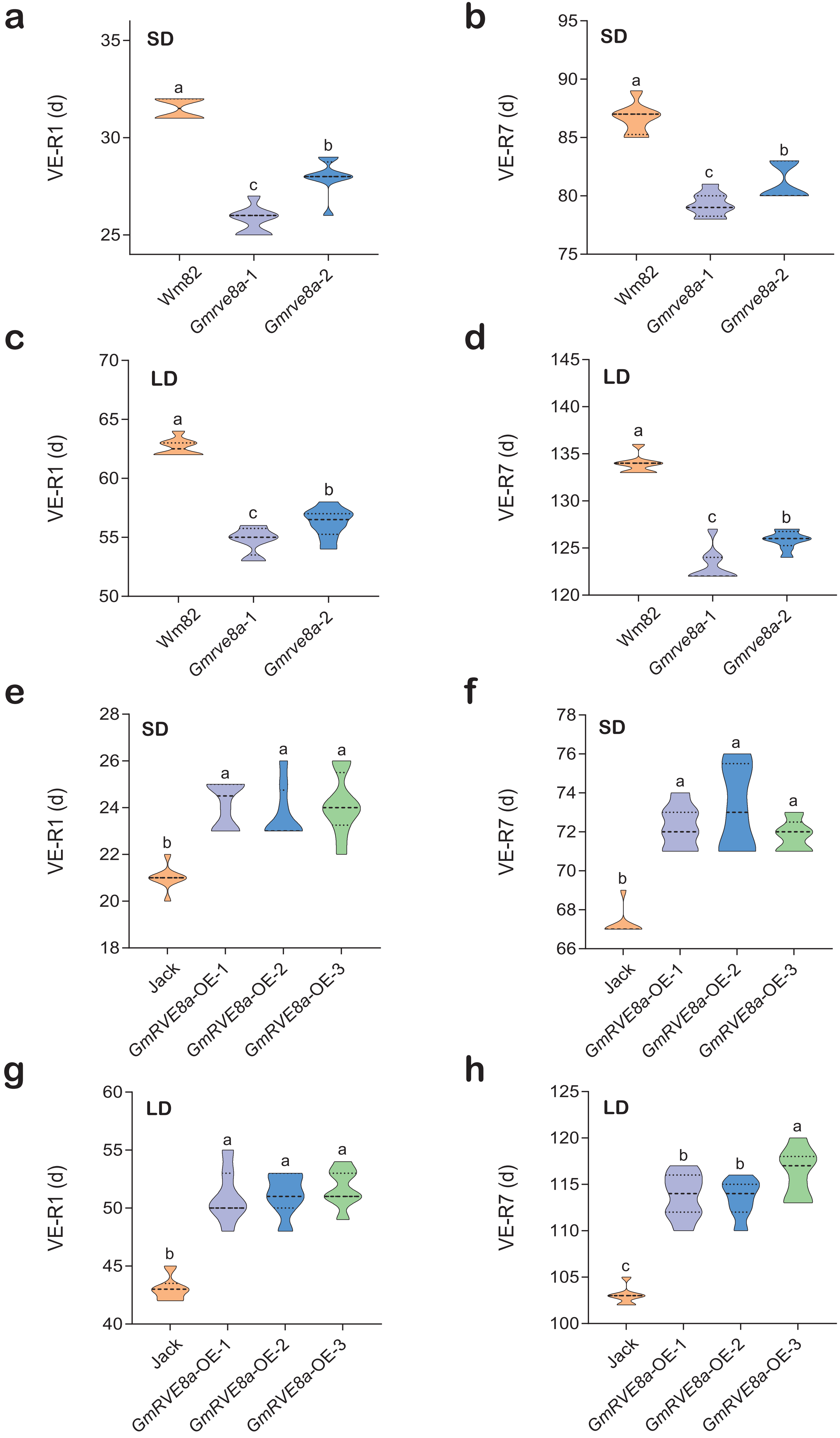
a
SD
VE-R1 (d)
35
30
25
a
c
b
Wm82
Gmrve8a-1
Gmrve8a-2
b
SD
VE-R7 (d)
95
90
85
80
75
a
c
b
Wm82
Gmrve8a-1
Gmrve8a-2
c
LD
VE-R1 (d)
70
65
60
55
50
a
c
b
Wm82
Gmrve8a-1
Gmrve8a-2
d
LD
VE-R7 (d)
145
140
135
130
125
120
a
c
b
Wm82
Gmrve8a-1
Gmrve8a-2
e
SD
VE-R1 (d)
28
26
24
22
20
18
b
a
a
a
Jack
GmRVE8a-OE-1
GmRVE8a-OE-2
GmRVE8a-OE-3
f
SD
VE-R7 (d)
78
76
74
72
70
68
66
b
a
a
a
Jack
GmRVE8a-OE-1
GmRVE8a-OE-2
GmRVE8a-OE-3
g
LD
VE-R1 (d)
60
55
50
45
40
b
a
a
a
Jack
GmRVE8a-OE-1
GmRVE8a-OE-2
GmRVE8a-OE-3
h
LD
VE-R7 (d)
125
120
115
110
105
100
c
b
b
a
Jack
GmRVE8a-OE-1
GmRVE8a-OE-2
GmRVE8a-OE-3
