# Supplementary material for: GmRVE8a, a circadian clock‐associated gene, negatively regulates flowering and maturity in soybean
Source: Plant J. 2026 Nov 4;128(3):e71205. doi: 10.1111/tpj.71205 (PMC13732551; doi:10.1111/tpj.71205)
Supplement: Supplementary file 9 [file TPJ-128-0-s012.pdf]

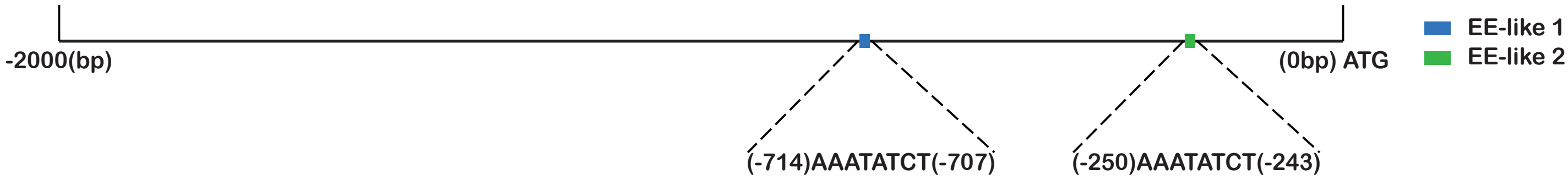

-2000(bp)
(0bp) ATG
EE-like 1
EE-like 2
(-714)AAATATCT(-707)
(-250)AAATATCT(-243)
