# Supplementary material for: GmRVE8a, a circadian clock‐associated gene, negatively regulates flowering and maturity in soybean
Source: Plant J. 2026 Nov 4;128(3):e71205. doi: 10.1111/tpj.71205 (PMC13732551; doi:10.1111/tpj.71205)
Supplement: Supplementary file 10 [file TPJ-128-0-s013.pdf]

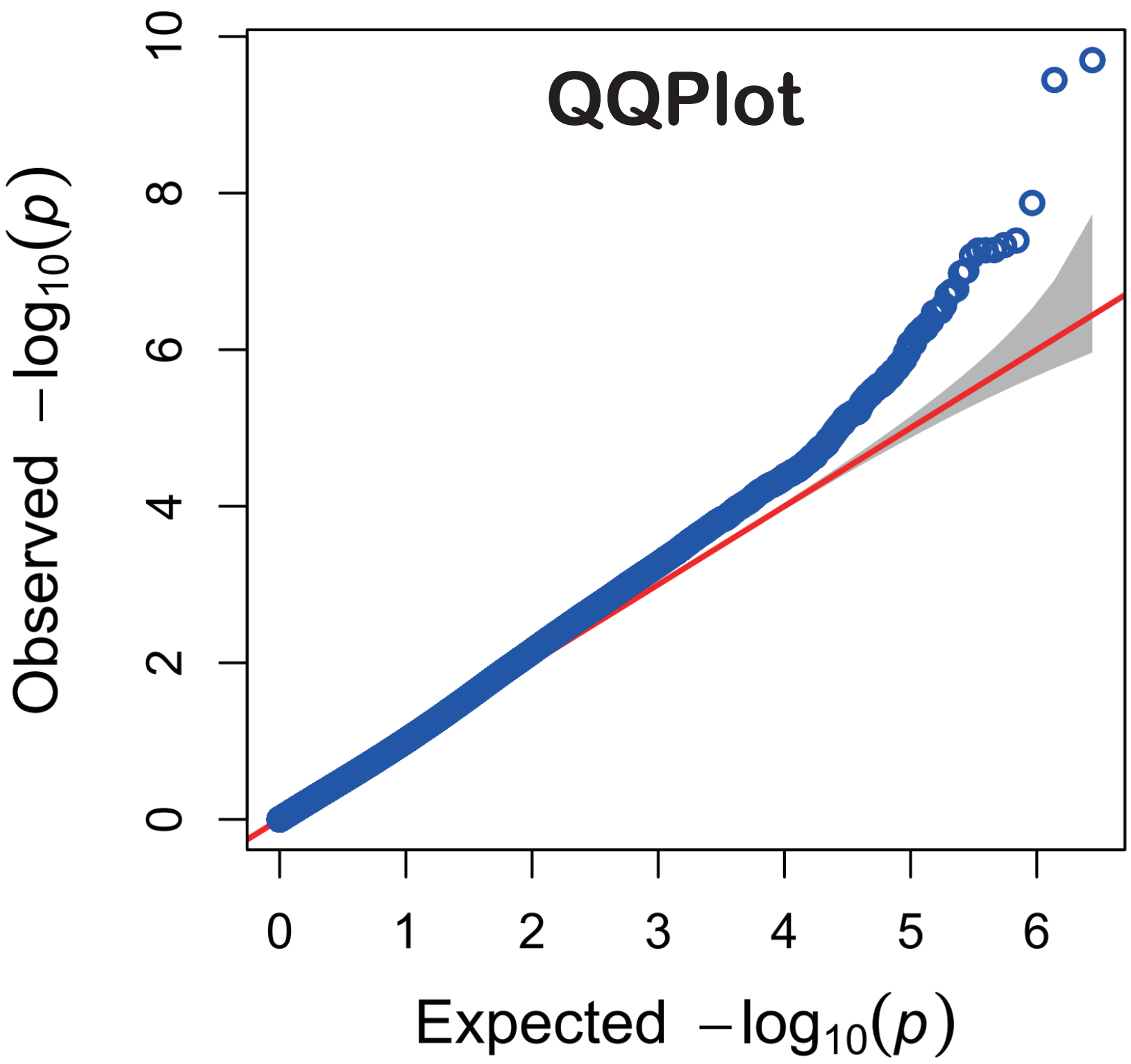

QQPlot
Observed −log10(p)
Expected −log10(p)
0
2
4
6
8
10
0
1
2
3
4
5
6
