# Supplementary material for: GmRVE8a, a circadian clock‐associated gene, negatively regulates flowering and maturity in soybean
Source: Plant J. 2026 Nov 4;128(3):e71205. doi: 10.1111/tpj.71205 (PMC13732551; doi:10.1111/tpj.71205)
Supplement: Supplementary file 11 [file TPJ-128-0-s009.docx]

**Table S2** Genomic positions of significant GWAS SNPs and corresponding candidate genes in 257 soybean accessions

| SNP | Chromosome | Position | *P* value | Candidate gene |
| --- | --- | --- | --- | --- |
| 1-6184373 | 1 | 6184373 | 8.21E-08 | unknown |
| 1-6516425 | 1 | 6516425 | 9.13E-08 | unknown |
| 2-11373248 | 2 | 11373248 | 8.46E-07 | unknown |
| 2-39026187 | 2 | 39026187 | 6.64E-07 | unknown |
| 3-31626046 | 3 | 31626046 | 8.96E-07 | *Glyma.03G177300* |
| 4-5823392 | 4 | 5823392 | 7.31E-07 | unknown |
| 4-50347838 | 4 | 50347838 | 7.46E-07 | unknown |
| 5-2521110 | 5 | 2521110 | 5.48E-08 | unknown |
| 5-9471708 | 5 | 9471708 | 9.84E-07 | unknown |
| 6-19011627 | 6 | 19011627 | 1.59E-07 | unknown |
| 6-40340818 | 6 | 40340818 | 4.66E-08 | unknown |
| 6-42105286 | 6 | 42105286 | 6.34E-08 | unknown |
| 6-43426650 | 6 | 43426650 | 6.02E-08 | unknown |
| 8-39636942 | 8 | 39636942 | 1.94E-07 | unknown |
| 8-46802248 | 8 | 46802248 | 4.75E-08 | unknown |
| 9-18682188 | 9 | 18682188 | 7.81E-09 | unknown |
| 9-48400335 | 9 | 48400335 | 9.84E-07 | unknown |
| 10-42463879 | 10 | 42463879 | 5.45E-07 | unknown |
| 11-10479784 | 11 | 10479784 | 9.27E-07 | unknown |
| 11-25123332 | 11 | 25123332 | 3.12E-10 | unknown |
| 11-28317220 | 11 | 28317220 | 9.38E-07 | unknown |
| 12-11294256 | 12 | 11294256 | 9.84E-07 | unknown |
| 12-21279458 | 12 | 21279458 | 7.36E-07 | unknown |
| 12-37717236 | 12 | 37717236 | 9.82E-07 | unknown |
| 13-35291977 | 13 | 35291977 | 7.81E-09 | unknown |
| 16-2016044 | 16 | 2016044 | 8.71E-07 | unknown |
| SNP | Chromosome | Position | P value | Candidate gene |
| 16-5704235 | 16 | 5704235 | 4.61E-07 | unknown |
| 16-35931476 | 16 | 35931476 | 3.82E-07 | unknown |
| 17-13417558 | 17 | 13417558 | 1.82E-07 | unknown |
| 17-37860156 | 17 | 37860156 | 9.25E-08 | unknown |
| 18-2948421 | 18 | 2948421 | 5.65E-07 | unknown |
| 18-25789729 | 18 | 25789729 | 5.85E-07 | unknown |
| 18-46369156 | 18 | 46369156 | 1.92E-07 | unknown |
| 18-51783500 | 18 | 51783500 | 3.79E-07 | unknown |
| 19-43684936 | 19 | 43684936 | 4.93E-07 | unknown |
| 19-43687363 | 19 | 43687363 | 6.83E-08 | *GmFT3b* |
| 19-43721425 | 19 | 43721425 | 4.36E-09 | unknown |
| 20-6846419 | 20 | 6846419 | 5.72E-07 | unknown |
| 20-8694249 | 20 | 8694249 | 9.84E-07 | unknown |
| 20-38952681 | 20 | 38952681 | 5.74E-07 | unknown |
