# Supplementary material for: GmRVE8a, a circadian clock‐associated gene, negatively regulates flowering and maturity in soybean
Source: Plant J. 2026 Nov 4;128(3):e71205. doi: 10.1111/tpj.71205 (PMC13732551; doi:10.1111/tpj.71205)
Supplement: Supplementary file 12 [file TPJ-128-0-s014.docx]

**Table S1** Sequencing summary of 257 re-sequenced soybean accessions

| Variety | Raw reads | Clean reads | Q30 (%) | Coverage (%) |
| --- | --- | --- | --- | --- |
| BeijiSoja 1 | 144259840 | 139509200 | 92.99 | 94.20 |
| BeijiSoja 6 | 141170042 | 133238934 | 92.72 | 94.27 |
| ChuangyeSoja | 139163838 | 135704926 | 92.83 | 92.68 |
| HeiheSoja | 81024410 | 80537234 | 92.62 | 91.44 |
| HumaSoja | 137372434 | 131494598 | 92.53 | 93.14 |
| PanguSoja | 143037110 | 138431612 | 92.52 | 94.20 |
| ZhangdiyingSoja | 138019666 | 134175904 | 93.06 | 93.60 |
| BayanSoja | 96565376 | 95991001 | 93.48 | 91.97 |
| BaiyangdianSoja | 99989020 | 99889850 | 93.75 | 92.99 |
| GuangxiSoja | 109704710 | 109592648 | 94.65 | 92.99 |
| Changjihuangdou | 74287416 | 61278178 | 94.21 | 94.60 |
| Taixingheidou | 82438073 | 78277944 | 90.64 | 93.02 |
| Miquanhuangdou | 79944212 | 76745029 | 95.6 | 91.80 |
| Fengchengniupidou | 78043148 | 69054875 | 95.52 | 94.15 |
| Fengdihuang | 129575816 | 127970030 | 95.4 | 94.34 |
| Huairouhuangdou | 72314554 | 65477950 | 92.62 | 94.90 |
| Weiqingdou | 73205471 | 71545847 | 90.26 | 93.21 |
| Heibiqing | 71582715 | 64690614 | 93.23 | 94.80 |
| Qunyingdou | 81745217 | 72072618 | 92.74 | 93.70 |
| Fengshouhuang | 82244572 | 82099825 | 92.3 | 93.88 |
| Naiyinheidou | 86098216 | 79235444 | 93.65 | 91.43 |
| Haiyangpamanqing | 94357882 | 94165060 | 93.15 | 94.99 |
| Liuyuehuang | 75584223 | 65733874 | 93.52 | 94.04 |
| Yunyizao | 84295194 | 82610304 | 92.52 | 91.54 |
| Chenliuniumaohuang | 70057168 | 64055126 | 90.37 | 92.17 |
| Baihuadou | 77719046 | 75044319 | 91.22 | 94.28 |
| Jinjiangdaqingren | 66106025 | 55231071 | 93.56 | 93.33 |
| Variety | Raw reads | Clean reads | Q30 (%) | Coverage (%) |
| Zigongqingpidou | 89384456 | 86537550 | 92.71 | 92.91 |
| Baishuidou | 75630473 | 67828108 | 93.17 | 92.30 |
| Yulindahuangdou | 60131690 | 55879386 | 95.87 | 91.36 |
| Suidaohuang | 80052173 | 70415954 | 94.44 | 91.67 |
| Juhuangdadou | 84886201 | 72327051 | 94.67 | 93.19 |
| Yangchunqingpidou | 76320310 | 63519864 | 94.35 | 92.8 |
| Wuhuasiyuehuang | 65014703 | 60869963 | 90.17 | 94.16 |
| Hengyangbayueqing | 74220272 | 51578176 | 94.51 | 93.13 |
| Jinningdahuangdou | 139389856 | 137480511 | 95.3 | 91.60 |
| Pingguohuangdou | 72913016 | 56175235 | 91.03 | 91.73 |
| Tongshanbopihuang | 82958522 | 60056228 | 96.8 | 91.96 |
| Huangfengwo | 74402344 | 31135582 | 94.16 | 92.65 |
| Shangraodaqingsi | 89372921 | 76062803 | 90.91 | 91.07 |
| Zigongdongdou | 72793110 | 24959538 | 94.89 | 92.53 |
| Lanxidaqingdou | 78476528 | 65357481 | 93.47 | 94.56 |
| Jiangledaqingdou | 61301904 | 38522928 | 94.71 | 91.89 |
| R-4 | 76493214 | 53978034 | 94.53 | 91.97 |
| Star 4-75 | 62428398 | 42977518 | 95.48 | 92 |
| Hujiao 07-2123 | 72231696 | 48878142 | 96.12 | 91.76 |
| Hujiao 07-2479 | 64082852 | 40782180 | 96.03 | 91.99 |
| Rassvet | 70398932 | 65173260 | 95.65 | 91.8 |
| Jug 30 | 74878914 | 44214760 | 96.43 | 92.06 |
| Mageva | 85243654 | 46870620 | 94.05 | 91.66 |
| R2 | 92676246 | 41789618 | 95.41 | 91.25 |
| Paula | 76368722 | 50375436 | 95.73 | 91.18 |
| OAC Vision | 88012858 | 40284004 | 96.42 | 91.41 |
| Maple Presto | 79851148 | 42743878 | 94.86 | 91.44 |
| Norman | 73726527 | 64104289 | 94.54 | 94.47 |
| Beihudou | 68130388 | 58010413 | 95.61 | 93.56 |
| Variety | Raw reads | Clean reads | Q30 (%) | Coverage (%) |
| Maple Ridge | 76673010 | 45618406 | 94.24 | 91.48 |
| Canatto | 75865442 | 44037064 | 95.07 | 91.42 |
| Jim | 81211874 | 71642408 | 96.17 | 90.89 |
| Daksoy | 73680606 | 45773816 | 96.11 | 91.03 |
| McCall | 85054832 | 81162843 | 92.26 | 91.04 |
| Glacier | 76832730 | 36715980 | 96.9 | 91.69 |
| Agassiz | 87475710 | 48875062 | 95.55 | 91.14 |
| Hidaka | 90933616 | 39112058 | 95.21 | 91.55 |
| Dongnong 41-C | 73379288 | 46076080 | 94.75 | 91.01 |
| Heihe 3 | 94646594 | 93880162 | 94.52 | 91.41 |
| Beifeng 2 | 73433578 | 73020144 | 92.15 | 92.53 |
| Heihe 9 | 78249630 | 77825182 | 92.07 | 94.25 |
| Huajiang 1 | 81878550 | 55369486 | 95.39 | 91.61 |
| Merit | 87729079 | 83172230 | 94.38 | 94.84 |
| Barnes | 80357014 | 36263306 | 95.9 | 91.48 |
| Heihe 51 | 82524426 | 82136040 | 93.78 | 93.22 |
| Norpro | 81141186 | 43437418 | 96.92 | 91.74 |
| Council | 73588324 | 32743780 | 95.62 | 91.35 |
| Prosoy | 91639564 | 46631668 | 94.85 | 91.69 |
| Sargent | 85119996 | 41176182 | 95.69 | 91.6 |
| Mengdou 30 | 96603592 | 96265052 | 95.26 | 91.44 |
| Chico | 101572886 | 23579806 | 95.73 | 90.71 |
| Clay | 85784876 | 81284213 | 94.23 | 92.14 |
| Wilkin | 60935437 | 57482352 | 91.12 | 93.75 |
| MN0201 | 77627626 | 50552686 | 96.53 | 91.62 |
| Simpson | 82562354 | 48318560 | 94.18 | 91.31 |
| Heihe 18 | 102829368 | 102420816 | 95.02 | 93.4 |
| Heihe 27 | 98599880 | 98198839 | 94.24 | 93.69 |
| Heihe 43 | 91143168 | 90847490 | 94.68 | 91.13 |
| Variety | Raw reads | Clean reads | Q30 (%) | Coverage (%) |
| Beidou 5 | 102866078 | 102539750 | 95.44 | 91.15 |
| Fengshou 10 | 64584580 | 64280228 | 92.21 | 91.72 |
| Hefeng 25 | 112282200 | 111882992 | 95.15 | 92.99 |
| Kexi 283 | 88388592 | 87929496 | 94.89 | 94.81 |
| Dawson | 74487144 | 57886396 | 94.63 | 91.49 |
| Beifeng 9 | 95074358 | 94572720 | 94.89 | 94.69 |
| Dongnong 72-806 | 65962195 | 56760792 | 94.49 | 94.52 |
| Fengshou 12 | 100582940 | 100079300 | 92.41 | 94.23 |
| Hefeng 35 | 88900962 | 88508178 | 94.25 | 92.11 |
| Dongnong 4 | 93690702 | 93405479 | 94.28 | 93.68 |
| NE1900 | 81847270 | 50067926 | 94.67 | 91.86 |
| Evans | 85334312 | 79808834 | 95.5 | 93.96 |
| Kato | 81561356 | 46200464 | 95.12 | 91.56 |
| MN1401 | 93552510 | 50478518 | 94.59 | 91.25 |
| Parker | 91376738 | 56738402 | 95.69 | 91.56 |
| Granite | 78327742 | 50078884 | 95.55 | 91.64 |
| Felix | 71028700 | 49192974 | 94.64 | 91.35 |
| Jilin 4 | 71012267 | 67146672 | 94.46 | 91.02 |
| Jiti 5 | 91463334 | 91204850 | 92.82 | 91.53 |
| Edou 8 | 146463036 | 144656915 | 95.59 | 92.61 |
| Suinong 14 | 105336940 | 104890338 | 95.53 | 91.46 |
| Heinong 35 | 93504754 | 92837450 | 95.17 | 91.15 |
| Suinong 8 | 87621752 | 87177087 | 94.92 | 91.41 |
| Hefeng 55 | 85912958 | 85684389 | 95.06 | 92.16 |
| Heinong 16 | 82452354 | 81864322 | 93.18 | 94.42 |
| Suinong 10 | 74850534 | 39365862 | 94.93 | 91.97 |
| Heinong 33 | 100612860 | 100091674 | 95.69 | 91.43 |
| Heinong 26 | 86225646 | 85764056 | 93.83 | 92.87 |
| Corsoy 79 | 86264410 | 43178144 | 95.94 | 91.32 |
| Variety | Raw reads | Clean reads | Q30 (%) | Coverage (%) |
| Hark | 93167725 | 89464571 | 92.9 | 91.06 |
| Vinton 81 | 68552078 | 47948174 | 95.66 | 91.36 |
| Olympus | 101869802 | 45700402 | 94.44 | 91.7 |
| Century 84 | 80835064 | 46197378 | 94.61 | 91.44 |
| IL1 | 77295604 | 51304876 | 95.58 | 91.8 |
| LN92-12054 | 60230068 | 30384974 | 95.14 | 91.66 |
| LN92-7369 | 80594622 | 43732182 | 96.26 | 90.96 |
| Iroquois | 75004286 | 39819784 | 94.42 | 91.86 |
| Jack | 64506696 | 41235722 | 96.86 | 91.12 |
| Harosoy63 | 95798917 | 88196155 | 95.79 | 94.01 |
| Amsoy | 90863070 | 90120272 | 94.29 | 91.84 |
| Tianlong 1 | 81797969 | 73109735 | 90.67 | 91.89 |
| Jidou 7 | 85685958 | 84831733 | 94.22 | 92.44 |
| Jilin 13 | 89880186 | 89644357 | 93.18 | 93.32 |
| Jilin 20 | 67242820 | 66814174 | 93.61 | 92.01 |
| Huangbaozhu | 93526418 | 93232495 | 92.79 | 92.15 |
| Jilin 3 | 99251342 | 98953276 | 93.32 | 92.76 |
| Jilin 30 | 80858364 | 80653720 | 93.17 | 94.76 |
| Zaofeng 1 | 80460886 | 69358770 | 94.86 | 93.95 |
| Jiyu 57 | 73759008 | 73551140 | 91.83 | 92.46 |
| Changnong 5 | 92887722 | 92690789 | 93.43 | 94.71 |
| Jiti 1 | 79289426 | 78999846 | 91.84 | 93.38 |
| Jiunong 22 | 101460472 | 100457864 | 93.65 | 94.23 |
| Fushou | 92500514 | 91573458 | 94.46 | 92.74 |
| Tiefeng 19 | 89156167 | 86215076 | 93.39 | 91.91 |
| Kaiyu 8 | 78582850 | 78348444 | 91.41 | 93.56 |
| Tiefeng 20 | 82193994 | 58861442 | 95.28 | 91.45 |
| Tiefeng 3 | 80624276 | 80023847 | 93.7 | 93.66 |
| Kaiyu 3 | 102413276 | 102093847 | 93.32 | 93.85 |
| Variety | Raw reads | Clean reads | Q30 (%) | Coverage (%) |
| Kaiyu 10 | 94081258 | 49140918 | 94.05 | 91.56 |
| Qihuang 10 | 66244112 | 52258074 | 96.51 | 91.67 |
| Ludou 11 | 134471462 | 127540606 | 77.7 | 92.98 |
| Jindou 3 | 76855634 | 73286351 | 94.88 | 94.55 |
| Jindou 2 | 89673695 | 86929980 | 90.04 | 94.68 |
| Dilworth | 63343314 | 38101566 | 95.49 | 91.49 |
| Dennison | 85405462 | 64235926 | 95.79 | 91.88 |
| Kottman | 83737750 | 71548304 | 96.97 | 91.5 |
| Corsica | 68940256 | 44228246 | 94.28 | 91.5 |
| LN89-5699 | 84102824 | 44846206 | 94.61 | 91.65 |
| Will | 95156696 | 56589348 | 96.03 | 91.58 |
| Wayne | 76289841 | 70803462 | 91.8 | 92.86 |
| IL2 | 81193452 | 47494058 | 96.66 | 91.65 |
| Lincoln | 85423606 | 63390828 | 94.71 | 91.34 |
| Fayette | 96143838 | 62924118 | 94.88 | 91.71 |
| Williams 82 | 67882018 | 45105426 | 96.76 | 91.44 |
| KS3494 | 67323224 | 54080404 | 95.76 | 91.62 |
| Zhechun 3 | 148584022 | 145856809 | 94.26 | 94.65 |
| Jindou 25 | 132950006 | 130899929 | 95.01 | 93.37 |
| Jindou 19 | 75886150 | 75411593 | 93.18 | 93.71 |
| Wenfeng 5 | 81297651 | 72257500 | 95.57 | 93.46 |
| Ludou 4 | 87049104 | 86858275 | 94.08 | 94.21 |
| Qihuang 34 | 78470814 | 69679920 | 95.72 | 91.45 |
| Yanhuang 1 | 79454882 | 79198512 | 90.5 | 94.13 |
| Hedou 13 | 154516738 | 152181477 | 95.12 | 92.4 |
| Dandou 4 | 81720085 | 75428141 | 93.74 | 92.89 |
| Jin 33 | 76620655 | 66645431 | 90.02 | 92.31 |
| Jin 8-14 | 77253003 | 65624996 | 90.84 | 92.54 |
| Liaodou 15 | 93662386 | 93430180 | 93.68 | 94.23 |
| Variety | Raw reads | Clean reads | Q30 (%) | Coverage (%) |
| Tiefeng 31 | 74459478 | 73830770 | 94.15 | 94.78 |
| Xiangdou 3 | 79367166 | 68459343 | 90.27 | 91.65 |
| Handou 5 | 119412564 | 117634539 | 94.89 | 94.48 |
| Jidou 12 | 138529966 | 136928984 | 94.67 | 91.87 |
| Jidou 17 | 128738222 | 127065657 | 95.56 | 93.97 |
| Guichun 1 | 122545604 | 121109567 | 95.34 | 93.53 |
| Zhonghuang 301 | 90821454 | 55258628 | 95.76 | 91.5 |
| Zhonghuang 35 | 78458008 | 77997614 | 94.47 | 92.2 |
| Zhonghuang 30 | 135144192 | 133139764 | 95.27 | 93.18 |
| Kefeng 6 | 123507932 | 117470305 | 94.93 | 91.98 |
| Zhongdou 19 | 155359418 | 148561389 | 95.14 | 94.92 |
| Zhonghuang 13 | 131625662 | 129873209 | 94.71 | 93.78 |
| Zhonghuang 37 | 145823036 | 143923346 | 95 | 94.94 |
| Zhonghuang 39 | 85383622 | 72937590 | 94.26 | 90.75 |
| UA 4805 | 88194838 | 32761174 | 95.09 | 91.51 |
| Lawrence | 89754036 | 62434032 | 95.09 | 91.59 |
| Omaha | 88197994 | 46336316 | 94.16 | 91.68 |
| Cutler | 83323670 | 63504908 | 93.22 | 92.33 |
| LS92-1800 | 84806662 | 56294120 | 95.29 | 91.53 |
| LD00-2817P | 79203008 | 64981412 | 96.58 | 91.44 |
| Calhoun | 64249124 | 47885826 | 95.65 | 91.9 |
| CF461 | 76071260 | 39187902 | 94.77 | 91.24 |
| Pennyrile | 79557170 | 44981738 | 96.3 | 91.75 |
| CF492 | 71507364 | 52460438 | 95.53 | 91.68 |
| 77-14 | 91674133 | 86930205 | 95.79 | 94.67 |
| Flyer | 96501162 | 52407636 | 96.76 | 91.21 |
| Youbian 30 | 82363886 | 82177715 | 92.32 | 92.29 |
| Guichun 8 | 144548978 | 142862104 | 95.39 | 93.14 |
| Qiandou 6 | 87323174 | 86405192 | 94.13 | 91.22 |
| Variety | Raw reads | Clean reads | Q30 (%) | Coverage (%) |
| Bahong 1 | 84973224 | 74288530 | 92.69 | 93.92 |
| Zheng 92116 | 133205536 | 131591242 | 95.06 | 92.56 |
| Xudou 5 | 82046655 | 70865241 | 91.65 | 94.11 |
| 58-161 | 67966186 | 58792010 | 90.31 | 93.2 |
| Tiefeng 29 | 84971230 | 84790886 | 93.41 | 91.4 |
| Dandou 1 | 85282910 | 79260673 | 91.31 | 93.11 |
| Yuejin 4 | 91711215 | 80230296 | 93.59 | 94.46 |
| Jindou 1 | 98466240 | 81862606 | 96.41 | 90.7 |
| Jindou 23 | 145555570 | 143163257 | 95.84 | 91.5 |
| Taiwan 75 | 146449288 | 144489666 | 95.3 | 92.76 |
| Hutcheson | 70879778 | 36860988 | 96.22 | 91.3 |
| Essex | 62714222 | 55110890 | 94.44 | 91.56 |
| Dare | 87213573 | 70143086 | 92.04 | 92.51 |
| Nathan | 84496442 | 40360902 | 96.62 | 91.36 |
| Bedford | 87593457 | 83851288 | 90.26 | 94.24 |
| R01-3474F | 67894466 | 63705466 | 94 | 90.44 |
| TN04-5321 | 64923316 | 43950882 | 94.62 | 92.36 |
| Edou 2 | 74177402 | 66023054 | 91.01 | 93.54 |
| Houzimao | 124822842 | 122911422 | 94.1 | 93.3 |
| TN03-350 | 85719240 | 57315930 | 96.19 | 92.7 |
| Dillon | 71154740 | 38308276 | 94.9 | 93.04 |
| Holladay | 73462428 | 54664898 | 96.42 | 92.86 |
| Hood | 72274730 | 62349455 | 91.2 | 92.9 |
| Tracy | 86735701 | 76395912 | 94.02 | 91.98 |
| Centennial | 83863261 | 76707002 | 92.33 | 94.87 |
| R01-2731F | 67204532 | 46185050 | 96.45 | 92.93 |
| Lonoke | 77539788 | 60041548 | 94.54 | 92.8 |
| Rhodes | 72739894 | 64492706 | 94.27 | 92.55 |
| NC-Roy | 72224068 | 52572226 | 96.34 | 92.84 |
| Variety | Raw reads | Clean reads | Q30 (%) | Coverage (%) |
| D95-6271 | 92665804 | 53723716 | 94.78 | 92.88 |
| G01-PR16 | 74589786 | 49575072 | 96.66 | 93.02 |
| Suxie 1 | 129287072 | 127797450 | 94.5 | 91.02 |
| Lee | 73014108 | 42733100 | 94.14 | 92.73 |
| Boggs | 73003552 | 26575548 | 94.98 | 92.61 |
| Braxton | 92021601 | 89742480 | 90.28 | 93.36 |
| Motte | 65147324 | 43093900 | 96.81 | 92.18 |
| Dowling | 53373602 | 28240044 | 95.29 | 94.4 |
| Benning | 79472230 | 61629410 | 94.4 | 92.47 |
| Prichard | 59114282 | 26353086 | 95.39 | 92.77 |
| Cook | 93968108 | 43484210 | 96.56 | 92.59 |
| Santee | 73541276 | 27330782 | 95.28 | 92.55 |
| Maxcy | 82535962 | 36715576 | 94.81 | 92.52 |
| Nandou 12 | 153557476 | 151764666 | 95.42 | 93.45 |
| Qiudou 1 | 70849233 | 62584194 | 92.53 | 93.85 |
| Jupiter | 82798858 | 62800268 | 94.11 | 94.03 |
| Alamo | 73209474 | 45620324 | 95.09 | 93.82 |
| Jupiter-R | 69621730 | 48139858 | 95.91 | 94.04 |
| FT-4 | 73197400 | 62213488 | 95.57 | 93.92 |
| FT-15 | 78353434 | 62330584 | 94.39 | 94.02 |
| UFV-3 | 66699456 | 46482110 | 94.87 | 93.98 |
| IAC-8 | 75050538 | 65923204 | 95.38 | 93.91 |
| UFV-8 | 64289944 | 47848076 | 94.03 | 94.08 |
| CIGRAS-51 | 87224142 | 80230574 | 96.42 | 93.33 |
| Huaxia 3 | 72483594 | 53922846 | 94.42 | 94.21 |
| CIGRAS-06 | 70844226 | 20306608 | 96.33 | 93.31 |
| I.C. 192 | 18274830 | 4070918 | 95.72 | 93.6 |
