# Supplementary material for: GmRVE8a, a circadian clock‐associated gene, negatively regulates flowering and maturity in soybean
Source: Plant J. 2026 Nov 4;128(3):e71205. doi: 10.1111/tpj.71205 (PMC13732551; doi:10.1111/tpj.71205)
Supplement: Supplementary file 13 [file TPJ-128-0-s015.docx]

**Table S3** Predicted genes in mapping region (38,989-kb to 39,087-kb) according to *Glycine max* Wm82.a2.v1 reference genome

| Gene ID | Function |
| --- | --- |
| *Glyma.03G177300* | *PROTEIN REVEILLE 8* |
| *Glyma.03G177400* | Large subunit ribosomal protein L4e (RP-L4e, RPL4) |
| *Glyma.03G177500* | APETALA2-LIKE PROTEIN 1 |
| *Glyma.03G177600* | REPEAT FAMILY PROTEIN, PUTATIVE, EXPRESSED-RELATED |
| *Glyma.03G177700* | TRANSCRIPTION FACTOR CBF_NF-Y_ARCHAEAL HISTONE DOMAIN-CONTAINING PROTEIN |
| *Glyma.03G177800* | Nucleic acid-binding, OB-fold // MCM OB domain |
| *Glyma.03G178000* | RIBOSOMAL PROTEIN S23 // 40S RIBOSOMAL PROTEIN S23-2 |
| *Glyma.03G178100* | INTERACTOR OF CONSTITUTIVE ACTIVE ROPS-LIKE PROTEIN |
| *Glyma.03G178200* | PROTEIN IQ-DOMAIN 2 |
